# The psychological subtype of intimate partner violence and its effect on mental health: a systematic review with meta-analyses

**DOI:** 10.1186/s13643-022-02025-z

**Published:** 2022-08-10

**Authors:** S. B. Dokkedahl, R. Kirubakaran, D. Bech-Hansen, T. R. Kristensen, A. Elklit

**Affiliations:** 1 Danish National Center of Psychotraumatology, Department of Psychology, Campusvej 55, 5230 Odense M, Denmark; 2 University of Southern Denmark, Odense, Denmark; 3 Prof. BV Moses Centre for Evidence-Informed Healthcare and Health Policy, Christian Medical College, Vellore, India; 4 Centre for Persons Subjected to Violence, Center of Social Medicine, Copenhagen University Hospital, Bispebjerg and Frederiksberg Hospital, Copenhagen, Denmark

## Abstract

**Purpose:**

The present study examines the association between psychological violence and posttraumatic stress disorder (PTSD), depression, and anxiety, while comparing the specific subtypes of psychological violence and simultaneously focusing on methodological shortcomings.

**Method:**

A systematic review and random-effects meta-analyses were applied on the three main outcomes: PTSD, depression, and anxiety. Four electronic databases were searched (PsycINFO, PubMed, EMBASE, and Web of Science), and a total of 194 studies were included (*k* = 149 for meta-analyses). GRADEpro was used to evaluate the certainty of the evidence from the meta-analyses.

**Results:**

Psychological violence had strong associations with the three main outcomes, with the strongest association for PTSD in both female and male victims. Coercive control was particularly associated with PTSD for female victims, while emotional/verbal and dominance/isolation had the strongest association with depression. Although the identified studies were characterized by gender bias, psychological violence appear to affect male mental health too.

**Discussion:**

Findings from the meta-analyses support the notion that psychological violence is a traumatic experience, which is strongly association with PTSD and other common mental health problems linked to trauma. GRADEpro rated the certainty of evince to be low, and thus, our confidence in the estimated effect is limited. Gender bias, the applied terminology, and other methodological shortcomings are discussed. Despite the substantial amount of research on this topic, more research is needed before we can draw any final conclusions on the effect of psychological violence on mental health.

**Supplementary Information:**

The online version contains supplementary material available at 10.1186/s13643-022-02025-z.

## Introduction

Psychological violence is the most common form of intimate partner violence (IPV). It is estimated to affect between 35 and 49% of men and women in Europe and the USA [1, 2]. Nevertheless, research has focused less on the independent effect of psychological violence on mental health, compared to physical and sexual IPV [3, 4]. Originally, researchers primarily considered psychological violence as a risk factor for later physical IPV [5–7], until qualitative interviews with female IPV victims, published in 1990, revealed that victims actually perceived psychological IPV to be worse than physical IPV [8, 9]. Since then, researchers have tried to conceptualize psychological violence and develop valid and reliable tools that can measure the phenomenon. The present systematic review concerns the mental health consequences associated with this specific subtype of IPV and a critical assessment of the current state of our knowledge.

It is well-documented that IPV can have severe mental health consequences for the individual. A meta-analysis by Golding [3] identified symptoms of posttraumatic stress disorder (PTSD) in 31–84.4% of IPV exposed women. Other comorbid mental health problems include substance abuse, depression, anxiety, sleep problems, and suicidality [10–12]. These studies primarily focused on physical IPV or pooled estimates of different subtypes of IPV. For the independent effect of psychological violence, most studies have examined the relationship with depression and anxiety [9, 12], and findings support an association [9, 12–14].

Although psychological violence has been recognized as harmful for more than three decades, the understanding of psychological violence as a traumatic event has been absent. This might be partly due to our understanding of what constitutes a trauma [9]. The first criterion of PTSD, Criterion A, in the Diagnostic and Statistical Manual of Mental Disorders (DSM) requires “exposure to actual or threatened death, serious injury, or sexual violence” for an event to constitute as a trauma [15]. However, the 11th edition of the International Classification of Diseases (ICD-11; [16] allows for a clinical evaluation of what constitutes a traumatic event for the individual, and thus, psychological violence could now be classified as such. A recent paper compared traumatic events defined by the DSM Criterion A with other traumatic non-Criterion A events [17], i.e., psychologically threatening events. Findings revealed that non-Criterion A psychologically threatening events, e.g., stalking or neglect, were as strongly associated with PTSD and complex-PTSD (C-PTSD), as common Criterion A events. More so, a multivariate analysis, controlling for sex and all other traumatic events, revealed that only the psychologically threatening non-Criterion A events were associated with PTSD and C-PTSD [17].

Physical and sexual IPV has long been a known risk factor for PTSD [3, 12, 18], and recently, a systematic review suggested that psychological violence is an independent risk factor for PTSD and other mental health outcomes, when controlling for the influence of other types of IPV [12]. This is consistent with Hyland et al. [17] and their results on psychologically threatening events. However, the systematic review did not conduct a meta-analysis [12], and thus, the strength of the relationship is unknown. The same applies to the outcome depression and anxiety [9, 12]. Moreover, it has not been examined how the varying subtypes and definitions of psychological violence affect the association with mental health, nor has the methodological shortcomings characterizing most research on IPV and psychological violence [19] been accounted for.

The conceptualization of psychological violence is characterized by a lack of a clear and consistent definition of the phenomenon; psychological violence, emotional abuse, non-physical violence, psychological aggression, and coercive control are just some of the terms used to conceptualize this IPV subtype. These discrepancies reflect basic variations in our understanding of the concept. Even the official organs defining IPV are not compliant in their definitions. The World Health Organization (WHO) separates psychological violence, i.e., “insults, belittling, constant humiliation, intimidation (e.g., destroying things), threats of harm, threats to take away children” ( [20]; p.1), from controlling behavior, “isolating a person from family and friends; monitoring their movements; and restricting access to financial resources, employment, education, or medical care” ( [20]; p.1), while the European Institute of Gender Equality state an overall definition, “Any act or behaviour which causes psychological harm to the partner or former partner. Psychological violence can take the form of, among others, coercion, defamation, a verbal insult, or harassment” ( [21]; p. 45). The lack of consensus in definitions presents a challenge when addressing psychological violence, as some researcher will apply a definition with severe controlling behaviors, while others will exclude these or study them separately. The present study will allow for the inclusion of all three definitions, as well as any other definition applied by researchers to compare the different subtypes and study their association with mental health [19].

The apparent challenges with the conceptualization of psychological violence stems from the continuum of abuse that characterize this type of violence. The continuum of psychological violence starts with what is typically referred to as “psychological aggression,” i.e., yelling and insults, and extends to what is commonly termed “coercion,” i.e., control and isolation. These variations are inevitably reflected in the measures used to screen for psychological violence and requires careful consideration when deciding who, where, and how to examine this issue. Otherwise, we risk equating verbal insults with threats to kill or take away children [19]. Other methodological challenges in current research include sampling, design, scoring method, and gender bias [9, 19]. For example, there may be qualitative and quantitative differences when comparing violent dating relationships with married or long-term cohabiting couples [9]. Just like culture might mediate the impact of IPV or influence how the violence is perceived and expressed [22, 23]. Moreover, convenience samples are not representative of IPV victims and cross-sectional research does not inform us about the causal effect of psychological violence on mental health. Most IPV measures have been developed based on research concerning female victims [9]. Hence, they may not be appropriate for studying male victimization [24]. Finally, scoring method (i.e., frequency vs. dichotomous scoring) influence the magnitude of the effect size, which is an important detail when comparing study results [9, 25]. When considering the psychometric measures used to evaluate psychological violence, there are significant differences in the varying use of subscales and definitions, which makes results difficult to compare and stresses the need to evaluate how these differences influence results [19]. This is further complicated when researchers use self-constructed questionnaires that lack systematic development, compared to valid and reliable measures [19, 25]. Further to this, dichotomous scoring carries the risk of undermining the systematic patterns of abuse, which is an important aspect of psychological violence.

Researchers have previously argued that PTSD should only be measured in relation to physical or sexual IPV [9]. However, our understanding of what constitutes a traumatic event has changed in recent years, and emerging evidence suggests that psychologically threatening events can have a severe impact on mental health [17]. Psychological violence is the most common IPV subtype [1, 2], and thus, it is paramount that we learn more about the mental health consequences associated with this type of abuse. Based on this, the present systematic review has three overall aims: [1] to estimate the independent association between psychological violence and three mental health outcomes, i.e., PTSD, depression and anxiety [2]; to examine the different subtypes of psychological violence and their independent associations with mental health; and [3] to conduct moderation analyses that accounts for some of the methodological challenges described above, i.e., to explore potential differences in the association across different samples, gender, culture, psychometric scales used to measure psychological violence, and study quality.

## Methods

A literature search was conducted to investigate the association between psychological violence and PTSD, depression, and anxiety. The present study is written in accordance with the Preferred Reporting Items for Systematic reviews and Meta-Analyses (PRISMA), and the study was originally registered in PROSPERO (#CRD42018116026). A protocol elaborating on the theoretical background and planned methodology was published before the search was conducted [19].

The research team behind this publication consist of experts on psychological violence (SBD and TRK), as well as PTSD and trauma symptomatology (SBD, TRK, and AE). An expert on systematic reviews (RK, co-author of protocol [19]) help design the study, and data-analyses were supported by a statistician with expertise in meta-analyses and health sciences (RK). Moreover, a research assistant assisted on the screening process in close collaboration with the team, and a librarian from the research institution assisted with the preparation of the search string.

The protocol for this systematic review outlines a more comprehensive study than what is presented here in the final manuscript [19]. Alterations were made since the extent of the literature far exceeded the expectations of the authors. In the following sections, we will explain how these alterations apply to the final study.

### Research question and outcome of interest

The central research question was formulated according to PICO (i.e., Population, Exposure, Comparison, Outcome), according to PRISMA: In adult men and women, who have been in a romantic relationship (P), and who have been exposed to psychological violence (I), how does this specific type of IPV and the different subtypes of psychological violence, e.g., emotional/verbal vs. dominance/isolation (C), affect mental health, especially trauma symptomatology (O)?

The study was concerned with the association between psychological violence and three main mental health outcomes, i.e., symptoms of PTSD, depression, and anxiety. These three outcomes were the basis for the meta-analyses, as originally planned. The protocol prepared for a qualitative synthesis on comorbid PTSD symptoms, defined by the National Institute for Health and Care Excellence (NICE) guidelines [19, 26]. However, due to the extent of the literature and the quality of most identified studies, the qualitative synthesis on comorbid symptoms was excluded from the final manuscript.

### Search method

**Fig. 1:**
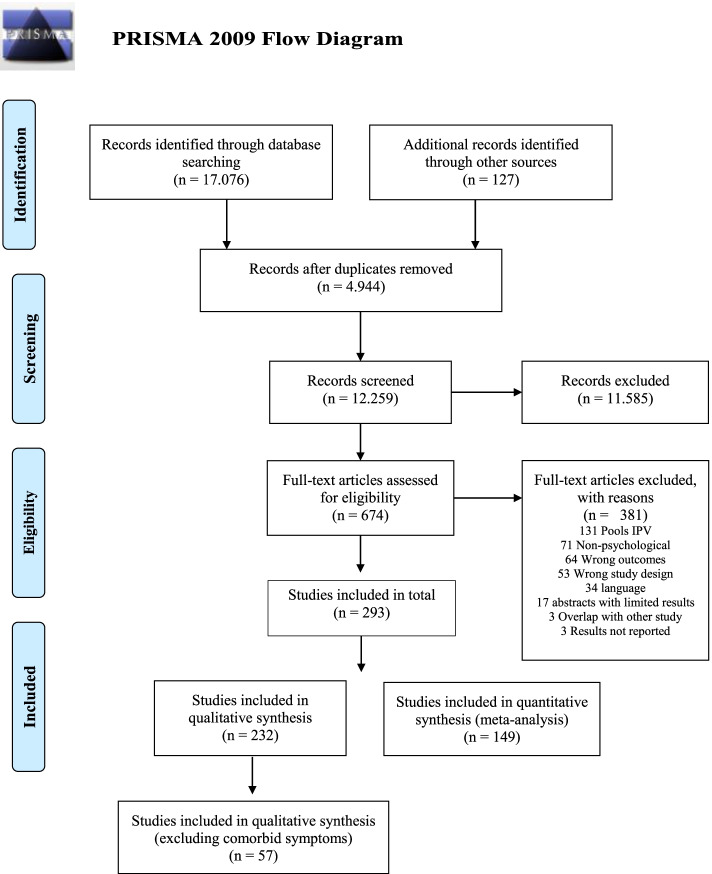
PRISMA 2009 Flow Diagram

Four electronic databases were searched: PsycINFO, PubMed, EMBASE, and Web of Science. Two researchers (SBD and DBH) completed a dual screening of title/abstracts in Endnote and included studies for full-text screening upon agreement. Additional records were further identified by hand-searching the two scientific journals, *Violence and victims* and *Journal of Interpersonal Violence*. These were searched from their first year of publication, the year 1986 for both journals. In case of any disagreement, a third researcher was included, and the matter was discussed until agreement was reached. Previous similar reviews and meta-analysis like Golding [3] and Lagdon et al. [12] were excluded due to discrepancies in study aims; however, references were examined for relevant studies. Initially, the authors planned to conduct the full-text screening and quality assessment as a dual process; however, due to the overwhelming amount of research, these final steps were only conducted by the first author in Covidence. No lower limit was defined for the time of publication. The earliest publication identified was from 1993 (See Appendix F). The initial search was conducted on January 15, 2019, and later updated on June 8, 2020. The final search is evident from Fig. 1. The full search string can be found in the published protocol [19] and was built on the following three components:

1. Population: The search was narrowed to partner-related violence with search terms such as *partner, spouse, marriage, domestic, dating, and dyads*.
2. Exposure: The search aimed to include all studies that examined some form of psychological violence, e.g., *psychological violence, emotional violence, psychological abuse, and coercive control.* This was supported by the inclusion of scales and measures developed for the purpose of measuring psychological violence, e.g., *CTS2, CAS, ISA-NP, and PMWI*.
3. Outcome: Included studies should contain mental health outcomes of which PTSD was the primary outcome. For this publication, comorbid mental health outcomes of depression and anxiety were included, based on the NICE Guidelines.

### Inclusion and exclusion criteria

Inclusion criteria were (I) exposure variable of psychological violence; (II) mental health outcome, i.e., PTSD, depression, and/or anxiety; (III) study subjects from an adult population (≥18) across different samples, e.g., dating, population survey, clinical settings, etc; and (IIII) peer-reviewed articles published in either English, German, Dutch, or any of the Scandinavian languages (i.e., Danish, Norwegian, or Swedish). The study excluded all qualitative studies as well as case studies, reviews, commentaries, editorials, letters to editorials, book chapters, and other non-primary research articles.

### Quality assessment

“The Qualitative Assessment Tool for Quantitative Studies” developed by the Effective Public Health Practice Project (EPHPP) was used to assess the quality of each individual study. The assessment is based on eight components: [1] selection bias, [2] study design, [3] confounders, [4] blinding, [5] data collection methods, and [6] withdrawals and dropouts, [7] intervention integrity, and [8] analysis [27] and has previously been used in research evaluating IPV psychometric tools [28]. For the present study, the seventh component of “Intervention integrity” was excluded, as it did not apply for the included studies. Based on the assessment, studies were classified as either weak, moderate, or strong (see Appendix A).

### Data extraction

Data was extracted in Excel by the first author. When reported, data extraction included the information on the following: author(s), year, sample size, population, country, age, gender, sexuality, study design, IPV assessment tool(s), mental health assessment, primary outcome (effect size), mean differences, secondary outcome(s), timeframe of assessment (lifetime or specified), scoring method(s), previous trauma, and previous mental health problems. Not all the extracted information was included in the final analyses due to the extensive amount of research and quality of the data.

### Data synthesis

Meta-analyses were applied to help estimate the common effect of psychological violence on mental health, i.e., PTSD, depression, and anxiety, by synthesizing individual results. A random-effects-model was conducted, as we expected high heterogeneity due to the large methodological variations in the included studies. A random effects meta-analysis assumes variance in the effect across different studies explained by real differences in effect, as well as by chance [19]. Heterogeneity is reported using the *I*^2^ statistic. The *I*^2^ statistic informs us what portion of the total variance in the effect size is caused by variance between the studies. As previously suggested, an *I*^2^ statistic of above 75% implies considerable heterogeneity, while an *I*^2^ statistic below 40% is not considered a concern [29]. Moderation analyses were applied to help reduce the heterogeneity by identifying confounding variables, i.e., psychometric measure, population, culture, subtypes of violence, and study quality.

All meta-analyses were conducted with the programming language R (RStudio). To pool effect sizes, all measures based on a continuous data (e.g., correlation coefficients, mean differences, ANOVA, *t* tests) were transformed into the effect size Hedges *g*. For odds ratios (OR), the log-OR with standard error (SE) was calculated for the meta-analysis using Review Manager 5.4. Separate meta-analyses were conducted for the Hedges *g* data and OR for the three mental health outcomes, PTSD, depression, and anxiety, and for the different gender groups (i.e., female, male, and combined^1^). Based on the available data, it was possible to compare three subtypes of psychological violence for the outcomes of PTSD and depression: emotional/verbal, dominance/isolation, and coercive control. These subtypes are commonly measured by different scales such as the Psychological Maltreatment of Women Inventory (PMWI) and the Coercion in Intimate Partner Relationships [30, 31].

All included studies are evident from Appendix A. As stated in the protocol, the study originally planned to conduct meta-regression to control for the influence of other types of IPV (e.g., physical or sexual) and previous trauma. Unfortunately, this was not possible from the extracted data. Studies that reported on the three main outcomes, PTSD, depression, or anxiety, but were not compliant for meta-analysis, are presented in a qualitative synthesis.

At a final step, GRADEpro was used to grade the certainty of the evidence and summarize the findings from the meta-analyses. GRADEpro assess the certainty of the evidence based on the following components: number of studies, study design, risk of bias, inconsistency, indirectness, and imprecision, with the possibility of adding other considerations (i.e., publication bias, large effect, plausible confounding, and dose response gradient), while also accounting for a summary of the findings. Based on this information, GRADEpro estimates the certainty of the evidence from *very low*, *low*, and *moderate* to *high*. This score reflects the extent to which we can be confident that the estimate of our effect size is correct. A high grade indicates that new research is unlikely to change our confidence in the estimate of our effect size. The GRADEpro assessment is evident from Table 3.

## Results

A total of *k* = 149 studies was included for meta-analysis (i.e., *k* = 68 for PTSD, *k* = 107 for depression, and *k* = 33 for anxiety). The total number of participants included for analysis were *N* = 229,762 (range 31–93,676; mean = 1542.03 [SD = 7905.70]), and the mean age of participants were 33.58 years (SD = 7.99). Most studies consisted of all-female samples (79.9%), compared with all-male (3.4%) and combined gender samples (16.8%). Some combined gender samples reported test-statistics for both the female and male participants, while other studies reported on the full sample. Most of the included studies were conducted in Europe (13.4%) or Northern America/Australia (AU; 63.1%), with the remaining studies reporting from South America (1.3%), Africa (10.1%), or Asian/Middle Eastern regions (12.1%). Finally, 89.8% of the included studies used a cross-sectional design, while 10.2% used a longitudinal design. Of the included studies, 51.0% were rated of weak quality, 38.9% were rated of moderate quality, while 10.1% were rated of strong quality, based on assessment tool by EPHPP [27]. All references included in meta-analyses can be found in Appendix F.

### Meta-analysis

#### PTSD

The meta-analyses of Hedges *g* on the association between overall psychological violence and PTSD found a large effect size for females (.90 [0.77; 1.04]), a medium effect size for males (.54 [0.41; 0.67]) and a large effect size for combined gender samples (.78 [0.35; 1.21]; Table 1).

**Table 1:** ***K*** = total number of studies; *studies reporting a combined score for males and females (e.g., LGBT-studies)

			PTSD					Depression					Anxiety			
		***K***	Effect	95% CI	***I***^**2**^	***τ***^**2**^	***K***	Effect	95% CI	***I***^**2**^	***τ***^**2**^	***K***	Effect	95% CI	***I***^**2**^	***τ***^**2**^
**Female victimization**																
***Hedges g***		45	0.90	[0.77; 1.04]	88%	0.1786, *p* < .01	57	0.70	[0.58; 0.81]	95%	0.1604, *p* < .01	20	0.58	[0.40; 0.76]	92%	0.1452, *p* < .01
***Odds ratio***		13	2.23	[1.37; 3.64]	95%	0.6428, *p* < .01	31	2.07	[1.61; 2.66]	97%	0.4460, *p* < .01	8	2.20	[1.75; 2.77]	62%	0.0577, *p* < .01
**Male victimization**																
***Hedges g***		4	0.54	[0.41; 0.67]	32%	0.0056, *p* = .22	8	0.43	[0.16; 0.69]	85%	0.1144, *p* < .01	3	0.27	[-0.02; 0.56]	67%	0.0434, *p* = .05
***Odds ratio***		–	–	–	–	–	6	2.42	[0.97; 6.02]	93%	1.1922, *p* < .01	3	4.21	[1.84; 9.64]	82%	0.0806, *p* < .01
Combined gender*																
***Hedges g***		3	0.78	[0.35; 1.21]	95%	0.1355, *p* < .01	8	0.45	[0.35; 0.54]	44%	0.0083, *p* = .08	2	0.22	[−0.15; 0.59]	85%	0.0603, *p* = .01
***Odds ratio***		–	–	–	–	–	4	2.38	[1.62; 3.50]	34%	0.0539, *p* = .21	3	1.67	[1.27; 2.21]	66%	0.0399, *p* = .05

A separate meta-analysis was conducted for odds ratio for female victims (OR = 2.23 [1.37; 3.64]; Table 1). It was not possible to conduct a meta-analysis on odds ratio outcomes for male samples.

Subgroup analyses were conducted on the female samples in respect to *Scale Measure, Population, Culture, Subtype of Psychological Violence, and Study Quality*; None of the subgroups helped reduce the heterogeneity and are thus supplied as Appendix B. Nevertheless, some subgroups did affect the effect size with, e.g., IPV samples and weak study quality demonstrating larger effect (Appendix B). For studies reporting on PTSD, the total number of participants were *N* = 30,783. The quality assessment rated 58.8% of weak quality, 33.8% of moderate quality, and 7.4% of strong quality.

#### Subtypes of psychological violence on PTSD

Comparing three subtypes of psychological violence (i.e., emotional/verbal, dominance/isolation, and coercive control), all subtypes revealed very high effect sizes for the association between each subtype and PTSD (Table 2).

**Table 2:** Female victimization across subtypes

		PTSD					Depression				
	Subtype	***k***	Hedges g	95% CI	***I***^**2**^	***τ***^**2**^	***k***	Hedges g	95% CI	***I***^**2**^	***τ***^**2**^
**Emotional/verbal**		8	0.91	[0.54; 1.29]	86%	0.2432, *p* < .01	9	0.91	[0.51; 1.31]	94%	0.3358, *p* < .01
**Dominance/isolation**		8	0.83	[0.49; 1.18]	87%	0.1991, *p* < .01	7	0.86	[0.52; 1.20]	90%	0.1764, *p* < .01
**Coercive control**		7	1.23	[1.05; 1.41]	53%	0.0280, *p* = .05	8	0.51	[0.35; 0.68]	45%	0.0235, *p* = .08

#### Qualitative synthesis of additional outcomes on PTSD

Twenty-one additional studies on PTSD were identified that could not be converted into Hedges *g* or odds ratio, e.g., regression coefficients, risk ratio, and difference in percentage. Of these, 16 studies demonstrated a positive association between psychological violence and PTSD [32–47], while the remaining five studies were insignificant [48–52].

#### Depression

The meta-analyses of Hedges *g* on the association between overall psychological violence and depression found a medium to large effect for females (.70 [0.58; 0.81]) and a medium effect size for males (.43 [0.16; 0.69]) and combined gender samples (0.45 [0.35; 0.54]; Table 1).

The meta-analysis for odds ratio revealed significant odds of depression for females (OR = 2.07 [1.61; 2.66]) and combined gender samples (OR = 2.38 [1.62; 3.50]), while the odds where slightly larger but insignificant for males (OR = 2.42 [0.97; 6.02]; Table 1).

The subgroups (i.e., *Scale Measure, Population, Culture, Subtype of Psychological Violence, and Study Quality*), again, did not help reduce heterogeneity. Some subgroups did, however, affect the effect size to some degree for both females and males (e.g., *Scale Measure;* Appendix C-D).

For studies reporting on depression, the total number of participants were *N* = 195,591. The study quality assessment deemed 47.7% of weak quality, 43.0% of moderate quality, and 9.3% of strong quality.

#### Subtypes of psychological violence on depression

The three subtypes of psychological violence (i.e., emotional/verbal, dominance/isolation, and coercive control), where also compared for the outcome of depression. Both emotional/verbal and dominance/isolation had large effect sizes for the association with depression (.91 [0.51; 1.31]; 0.86 [0.52; 1.20], respectively). The association between coercive control and depression showed a medium effect size (.51 [0.35; 0.68]; Table 2).

#### Qualitative synthesis of additional outcomes on depression

Another 37 studies examined the association between psychological violence and depression; twenty-nine of the identified studies found a significant association [13, 34, 35, 39, 42, 45–47, 49, 53–73], while five studies found a non-significant association [50, 74–77]. Three studies did not report test-statistics [46, 78, 79]. For Hazen et al. [61] and Lawrence et al. [13] significant findings varied across subtypes.

#### Anxiety

The meta-analysis of Hedges *g* on the association between overall psychological violence and anxiety found a medium effect size for female victims (.58 [0.40; 0.76]) and a small effect size for males (0.27 [−0.02:0.56]) and combined gender samples (.22 [−0.15; 0.59]).

The meta-analysis on odds ratio revealed larger odds of anxiety for males (OR = 4.21 [1.84; 9.64]) compared to females (OR = 2.20 [1.75; 2.77]) and the combined gender samples (OR = 1.66 [1.08; 2.57]).

Again, subtype analyses were conducted for females on *Scale Measure, Population, Culture* and *Study Quality*, which did not help reduce the heterogeneity, although some subgroups did affect the effect size (Appendix E). Unfortunately, there was not enough data available to compare subtypes of psychological violence for the anxiety outcome. For studies reporting on anxiety, a total of *N* = 53.286 participants was represented. Quality assessment deemed 45.5% of weak quality, 42.4% of moderate quality, and 12.1% of strong quality.

#### Qualitative synthesis of additional outcomes on anxiety

Finally, thirteen studies found a significant association between psychological violence and anxiety [13, 38, 40, 49, 59, 63, 65, 68, 78, 80–83], while three studies found an insignificant association [61, 76, 84]. For Hazen et al. [61], the association was found to be insignificant after controlling for physical and sexual violence, and Lawrence et al. [13] found the association to be significant only for some subtypes for both husband and wives. Lastly, three studies found psychological violence to predict a combined depression/anxiety outcome [85–87].

#### GRADEpro

Results from the meta-analyses on female victimization are presented in the GRADEpro table for PTSD, depression, and anxiety (Table 3). As evident from the table, results are graded primarily with “low certainty of evidence,” excepts for the OR-outcome on PTSD, which is graded as “very low certainty of evidence.” Thus, our confidence in the effect estimate is limited. The low certainty evidence is largely explained by the applied methodology of the included studies, which is primarily observational studies. This was also reflected in the high heterogeneity identified by the meta-analyses.

**Table 3:** GRADEpro

Summary of findings						
Psychological violence on mental health						
Population: Females from varying samples Setting: Primarily observational studies. Exposure: Psychological violence Outcome: PTSD, depression and anxiety						
Outcomes	Anticipated absolute effects^*^ (95% CI)		Relative effect (95% CI)	№ of participants (studies)	Certainty of the evidence (GRADE)	Comments
	Risk with [comparison]	Risk with [intervention]				
PTSD (Hedges g) Assessed with: multiple validated scales	0.90 (0.77; 1.04 95% CI)		-	8393 (45 observational studies)	⨁⨁◯◯ LOW ^a,b,c^	
PTSD (odds ratio) Assessed with: varying types of measures	**Study population**		**OR 2.23** (1.37 to 3.64)	15,796 (13 observational studies)	⨁◯◯◯ VERY LOW ^c,d,e^	The World Mental Health Survey has examined the 12-month prevalence of cross-country PTSD. The prevalence varied significantly by country income, with lower-low middle-income countries demonstrating a prevalence of 1.5% compared with 3.6% in high-income countries [88]. This variation is translated for the anticipated absolute effect comparing study population with low and high prevalence variations for PTSD.
	10 per 1000	**22 per 1000** (14 to 35)				
	**Low**					
	15 per 1000	**33 per 1000** (20 to 53)				
	**High**					
	36 per 1000	**77 per 1000** (49 to 120)				
Depression (Hedges g) Assessed with: multiple validated scales	0.69 (0.58; 0.81 95% CI)		-	112,487 (56 observational studies)	⨁⨁◯◯ LOW ^b,d^	
Depression (odds ratio) Assessed with: various types of measures	**Study population**		**OR 2.13** (1.54 to 2.95)	74,147 (30 observational studies)	⨁⨁◯◯ LOW ^e,f^	Kessler and Bromet [89] have reviewed the 12-month prevalence estimate of Major Depressive Disorder in 18 World Mental Health Surveys. The prevalence estimates ranges from 2.2 to 10.4% across the 18 countries. This variation is translated for the anticipated absolute effect comparing study population with low and high prevalence variations for depression.
	10 per 1000	**21 per 1000** (15 to 29)				
	**Low**					
	22 per 1000	**46 per 1000** (33 to 62)				
	**High**					
	104 per 1000	**198 per 1000** (152 to 255)				
Anxiety (Hedges g) Assessed with: multiple validated scales	**0.58** (0.4; 0.76 95%CI)		-	7339 (20 observational studies)	⨁⨁◯◯ LOW ^b,g^	
Anxiety (odds ratio) Assessed with: various types of measures	**Study population**		**OR 2.20** (1.75 to 2.77)	37,814 (8 observational studies)	⨁⨁◯◯ LOW ^e,h^	Baxter, Scott, Vos, and Whiteford [90] applied a meta-regression of prevalence studies of anxiety disorders from 44 countries. The estimated adjusted prevalence varied from 7.6 to 17.7% within the past 12 months across countries. This variation is translated for the anticipated absolute effect comparing study population with low and high Prevalence variations for anxiety.
	10 per 1000	**22 per 1000** (17 to 27)				
	**Low**					
	76 per 1000	**153 per 1000** (126 to 186)				
	**High**					
	177 per 1,000	**321 per 1000** (273 to 373)				

*The risk in the intervention group (and its 95% confidence interval) is based on the assumed risk in the comparison group and the relative effect of the intervention (and its 95% CI)

*CI* confidence interval, *OR* odds ratio

GRADE Working Group grades of evidence

High certainty: We are very confident that the true effect lies close to that of the estimate of the effect

Moderate certainty: We are moderately confident in the effect estimate: The true effect is likely to be close to the estimate of the effect, but there is a possibility that it is substantially different

Low certainty: Our confidence in the effect estimate is limited: The true effect may be substantially different from the estimate of the effect

Very low certainty: We have very little confidence in the effect estimate: The true effect is likely to be substantially different from the estimate of effect

Explanations

^a^. *I*^2^ statistic of 94%

^b^. E.g., gender bias, convenience samples, design

^c^. Large range in confidence interval—imprecise results

^d^. *I*^2^ statistic of 95%

^e^. Design, measures, sampling

^f^. *I*^2^ Statistic of 97%

^g^. *I*^2^ Statistic of 91%

^h^. *I*^2^ Statistic of 62%

#### Heterogeneity

All meta-analyses revealed high heterogeneity. This was expected due to the variety of studies included and is not considered a concern for the interpretation of the results. Moderation analyses were conducted for all outcomes concerning potential risks of bias (i.e., the psychometric measure, population, culture, subtype of violence, and study quality); however, these were not found to reduce the heterogeneity considerably and overall did not affect the results (Appendix B, C, D, E).

## Discussion

Although researchers have previously argued that that PTSD should only be measured in relation to physical or sexual IPV [9], our understanding of what constitutes a traumatic event has changed in recent years with emerging evidence suggesting that psychologically threatening events can have a severe impact on mental health, including PTSD symptomatology [17]. Moreover, psychological violence is estimated to be the most common type of IPV [1, 2], and thus, it is paramount that we understand the consequences that this type of abuse can have on victims. Therefore, the primary aim of the present study was to examine the association between psychological violence and the three mental health outcomes: PTSD, depression, and anxiety.

As evident from the result section, findings from the random effects meta-analyses revealed strong association between psychological violence and all mental health outcomes, although the strength of the associations varied across the difference outcomes, subtypes, and gender. Overall, psychological violence had the strongest association with PTSD for both female and male victims, compared with depression and anxiety. This finding is in accordance with previous research on the association between IPV and PTSD [3, 12]. Although the effect sizes of Hedges *g* and OR can be difficult to compare, outcomes based on continuous data and reported as Hedges *g*, appeared to generate larger effect sizes compared with dichotomous scoring, reported as OR [91]. This is in line with previous research suggesting that scoring method influence the magnitude of the effect size [25]. Interestingly, for female victims, the different subtypes of psychological violence appear to influence symptoms of PTSD and depression differently to some extent. The subtype of coercive control had the largest effect on PTSD (Hedges *g* = 1.23), although both emotional/verbal and dominance/isolation had large effect sizes for PTSD as well (Hedges *g* = .91 and .83, respectively). Results were somewhat different for depression where both emotional/verbal (Hedges *g* = .91), and dominance/isolation (Hedges *g* = .86) had large effect sizes, whereas coercive control only had a medium effect size for the association with depression (Hedges *g* = .51; See Table 2). Unfortunately, there was not enough data to study the different subtypes and their association with anxiety, nor to study the associations for male victims.

Although this study did not estimate the cause and effect between psychological violence and mental health, it demonstrates that psychological violence is strongly associated with mental health problems that are typically linked to trauma. This supports recent findings from studies that examined the association between adverse childhood events and trauma symptomatology, as part of the emerging evidence for the revision for the ICD-11, including the revision of PTSD, C-PTSD, and a new trauma definition [16]. Findings revealed significant associations between traumatic non-Criterion A events in childhood and later PTSD and C-PTSD symptomatology [92, 93], which is consistent with what Hyland et al. [17] found in an adult nationally representative sample from Ireland. Hyland et al. [17] argued that these findings do not suggest that PTSD and C-PTSD do not occur as a response to a traumatic event. Instead, it emphasizes that what constitutes as a traumatic event cannot be delimited to physical or sexual threats that appear traumatic not only for the victim, but for people in general. Thus, a traumatic event might be more accurately defined by the feelings of threat or horror it can cause the individual [17]. Psychological violence can be both latent and subtle and therefore difficult to express for the individual. Moreover, it is rarely characterized by a single event but represents repeated and prolonged victimization that might be extremely threatening or horrific for the individual. Findings from the present study supports the notion that psychological violence should be studied in association with PTSD, as there appears to be strong associations between the different subtypes of psychological violence and mental health, including PTSD.

Another important finding from this study is the disclosure of the varying terminology used to label psychological violence, as evident from Appendix A. Multiple different labels were used with the most common being some form of psychological violence (e.g., psychological violence, abuse, victimization), followed by emotional abuse and aggression. Yet, labels such as harassment, behavioral abuse, and verbal abuse were also identified. Some studies reported different subtypes and thus used different labels, while other studies used different labels interchangeably to describe psychological violence as a general phenomenon. It is paramount that we reach a common understanding and adapt this understanding to our language; otherwise, we risk undermining the understanding of the effect psychological violence has on mental health. The major discrepancy in our language further complicates our ability to communicate what psychological violence is to the public. We cannot expect the general population to fully understand and respect the gravity of psychological violence until IPV researchers and clinical practitioners reach a common understanding. For exploratory reasons, this study used a broad definition, on which the systematic search was based. This definition includes “any act or behavior which causes psychological harm” (EIGE, 2017; p. 45), which refer to the *effect* of psychological violence rather than the *act* of violence itself. Such definitions should be avoided for research purposes in the future, as it inevitably interferes with the study of cause and effect.

### Strengths and limitations

Despite the overall strength of meta-analyses and their ability to derive a pooled estimate of the true effect, the current study has several important limitations. Due to the nature of most IPV research, which uses cross-sectional designs, findings from the current study do not inform us about the cause and effect between psychological violence and mental health. Instead, this study merely examines the strength of the association between psychological violence and the mental health outcomes. Furthermore, meta-regression would be useful to control for other types of IPV that often overlaps with psychological violence, as well as previous trauma and previous mental health problems. Unfortunately, the heterogeneity of the data did not allow for such an analysis. Overall, the large heterogeneity represents a limitation in this study as well as in IPV research in general. This is likely explained by the varying samples, design, scales, and overall methods applied. This merely supports the authors initial notion regarding the lack of a clear and consistent definition of psychological violence. Moreover, most of the included studies were rated as low-quality research. These studies were included due to the explorative nature of this systematic review. Nevertheless, they should be considered a limitation (see Appendix B, C, D, E for subgroup analyses controlling for study quality). The outcomes examined in the current study were merely symptom-based (e.g., symptoms of PTSD, depression, anxiety). For a more accurate estimate, future research should examine the association between psychological violence and mental health based on adequate psychiatric assessment with a clear definition of the applied diagnostic criteria (i.e., DSM-5 or ICD-11). Finally, this study merely included primary research articles, as explained in the exclusion criteria. This inevitably means that important contributions, e.g., dissertations, reports, and unpublished documents, is missing, which might affect the results.

Despite the important limitations, which primarily originates from the quality of the included studies and thereby the certainty of the evidence, the present study also has noteworthy strengths. First and foremost, the study expands our understanding of psychological violence as a traumatic event and evaluates how the different subtypes of psychological violence are associated with mental health differently. Secondly, the comprehensive amount of research included for meta-analyses attests to the power of the study, despite of the low certainty of the evidence. Thirdly, GRADEpro, which is a recognized grading tool used to develop clinical guidelines in health care, was used to estimate the certainty of the evidence. GRADEpro helped determine the low certainty of the evidence despite the strong associations identified by meta-analyses. Finally, the present study included male victims, which otherwise have been neglected in past IPV research [94].

### Future research

This study examined the association between psychological violence and the three mental health outcomes. To estimate the causal effect between these factors, more longitudinal studies are needed, which controls for the influence of previous mental health problems and other types of abuse and studies the long-term mental health consequences.

Considerable gender bias was disclosed in the present study. As stated in the introduction, population-based surveys have demonstrated that a high prevalence of both men and women are exposed to psychological violence [2, 95]. Nevertheless, most studies identified for meta-analysis only examined the association between psychological violence and mental health in female victims, with only a few studies including males. Yet, findings suggested a significant association with a medium effect size for PTSD and depression in male participants. This association was smaller than what was found for female victims; however, this is not surprising seeing that women are more likely to develop PTSD in general, compared to men [92, 96]. Moreover, researchers have previously argued that psychological violence targeting men might be different from psychological violence targeting women [97, 98], and McHugh and colleagues [24] have argued that most validated measured used to screen for psychological violence have been developed based on female experiences with this type of abuse. It is critical for future research to focus on male victims when considering the mental health consequences following psychological violence and IPV in general. This notion is further stressed by Hyland et al. [17] who found psychologically threatening events to be a predictor of both PTSD and C-PTSD, after controlling for gender and the influence of other traumatic events. Although the psychologically threatening events were not particularly associated with partner abuse in that study, it suggests that psychologically threatening events are traumatic for male victims too and should be acknowledged as such [17].

In addition to an increased focus on gender bias in IPV research, more studies should examine psychological violence and how it can have specific expressions in certain stages of life or for certain minority groups. For instance, reproductive coercion before or during pregnancy is associated with other types of IPV and represents a specific type of control in which the partner pressures or threats to become pregnant, try to control the pregnancy outcome (i.e., termination or continuation), or conduct birth-control sabotage. This specific type of coercion has also been associated with mental health problems [99, 100]. These tactics, however, overlap with common features of psychological violence, and thus, it would be beneficial to study the unique patterns of reproductive coercion and how it affects mental health independently. Another important example is violence against LGBT+ individuals, who might be slightly more exposed to IPV than their heterosexual peers [101, 102]. Again, LGBT+ individuals may experience unique forms of psychological violence or identity abuse, e.g., threatening to “out” a person’s sexuality to their family or workplace before they themselves choose, which require us to be aware of unique patterns that may be specific for certain groups [25, 103]. A final example is ethnic minority individuals who experience negative social control and honour-related conflicts within their family (e.g., forced marriage, monitored at school or on social media, or threat of re-education trips out of the country [104];). Again, this type of abuse resembles that of psychological violence and are likely to occur from early stages of life and into marriage. It affects not only the relationship with an intimate partner, but with the entire family. Awareness of the specific dynamics surrounding psychological violence and how it affects mental health can have important clinical implications regarding detection, prevention, and treatment for the victims.

As stated in the introduction, psychological violence is the most common form of IPV [1, 2]. However, these estimates are limited to Europe and the USA, and although similar findings have been reported in other cultural settings, including China [105], Bangladesh [106], and sub-Saharan Africa [107], more research is needed on cultural differences in the prevalence and expression of psychological violence across different countries, and how these differences affect mental health. The present study tried to address this through subgroup analyses, but due to high heterogeneity results were only reported in the supplementary materials (Appendix  B, C, D, E).

### Clinical implications

Psychological violence should be recognized and taken just as seriously as physical and sexual violence. The public, practitioners, and authorities should be made aware of this type of violence and the severe mental health consequences that can follow. This includes the recognition of psychological violence as a potentially traumatic event, which could be a risk factor for PTSD and other commonly related mental health problems.

It is important that practitioners who meet victims of IPV are aware of the different dynamics and behaviors that defines and constitutes the construct of psychological violence to understand the victims’ experiences and to avoid minimizing the severity of the abuse. For instance, verbal and emotional abuse may appear to be less severe when compared with coercive control, or physical or sexual violence; however, this study demonstrated a strong association with both PTSD and depression for this specific subtype of psychological violence.

Finally, the public, practitioners, and authorities should also be informed about male victimization. It is important to recognize that psychologically threatening events can have negative mental health consequences for male victims too [17, 92, 93], including psychological IPV, and thus, preventive efforts and treatment interventions should also be available for male victims.

## Conclusion

In conclusion, psychological violence had strong associations with mental health problems like PTSD, depression, and anxiety. Therefore, psychological violence should be acknowledged as a severe form of IPV equal to physical and sexual violence. These findings lend support to the notion that psychologically traumatic events are important risk factors for PTSD. Coercive control was particularly associated with PTSD for female victims, while emotional/verbal and dominance/isolation had stronger associations with depression. Research on male victimization is scarce and thus more research should focus on this. Despite the comprehensive amount of research on this topic, the certainty of the evidence is low, as evident from the GRADEpro in Table 3. This is explained by the methodological challenges that IPV research currently face, i.e., cross-sectional data, sampling, and design. Furthermore, a clear and consistent definition of psychological violence is currently lacking in IPV research, and this includes the use of valid and reliable tools for such a complex phenomenon. Thus, more high-quality research is needed before we can draw any final conclusions. This is a critical call for future research to carefully consider the applied methodology when conducting IPV research.

## Supplementary Information

**Additional file 1: Appendix A.** All included studies.

**Additional file 2.** PTSD Subtype Analyses – Female Victimization

**Additional file 3.** Depression Subtype Analyses – Female Victimization

**Additional file 4.** Depression Subtype Analyses – Male Victimization

**Additional file 5.** Anxiety Subtype Analyses – Female Victimization

**Additional file 6.** References Used in Meta-Analyses

## Data Availability

Not applicable.

## Footnotes

1 Some studies report the association for a mixed gender sample, e.g., university students or sexual minority groups with LGBT+ individuals.
