# Supplementary material for: The psychological subtype of intimate partner violence and its effect on mental health: a systematic review with meta-analyses
Source: Syst Rev. 2022 Aug 10;11:163. doi: 10.1186/s13643-022-02025-z (PMC9364557; doi:10.1186/s13643-022-02025-z)
Supplement: Supplementary file 1 — Additional file 1: Appendix A. All included studies. [file 13643_2022_2025_MOESM1_ESM.docx]

Appendix A. All included studies.

| **Authors** | **N** | **Country** | **Age** | **G*** | **Population** | **DE** | **IPV Scale(s)** | **Outcome(s)** | **Terminology** |
| --- | --- | --- | --- | --- | --- | --- | --- | --- | --- |
| Ahmadabadi, 2020 | 1528 | Australia | 20.6 (.9) | F/M | Prospective birth cohort | LS | CAS, the Emotional Abuse Scale | Depression, anxiety | Emotional abuse |
| Alhalal, 2019 | 299 | Saudi Arabia | 36.11 | F | Married women from nine primary health care centres | CS | CAS, the Emotional Abuse Scale | PTSD, depression | Emotional abuse |
| Al-Modallal, 2012a | 267 | Jordan | 31 (8.2) | F | Refugee camps | CS | WHO domestic violence measure (controlling) + self-con emotional abuse | Depression, anxiety, stress | Psychological violence: Emotional and controlling behaviour |
| Al-Modallal, 2012b | 101 | Jordan |  | F | Educated women | CS | Self-con: physical and emotional | Depression, stress, self-esteem | Harassment |
| Albright, 2019 | 2658 | US | 30.2 (10.5) | F/M | NCHA II; College students; who served in the U.S. Armed Services | CS | Inspired by the Centres for Disease Control and Prevention (1-item per); Physical, emotional, and sexual | Depression, anxiety, alcohol, drugs, suicide, anorexia | Emotional abuse |
| Ali, 1999**^+^** | 40 | Canada | 34.7 (8.7) | F | Women from therapy centre | CS | Emotional abuse interview (modified of LEDS), The Brown and Harris methodology (modified) | Depression | Emotional abuse |
| Arias, 1997 | 66 | US | 26.9 (7.1) | F | Community couples | CS | CTS | Depression | Psychological aggression |
| Arias, 1999**^+^** | 68 | US | 36 | F | Women shelter | CS | CTS, PMWI | PTSD | Psychological abuse |
| Avant, 2011**^+^** | 191 | US | 19.7 (1.6) | F/M | College students | CS | CTS2 | PTSD | Psychological abuse |
| Babcock, 2008 | 202 | US | 30 | F | Community Couples in conflict | CS | CTS, The emotional abuse scale | PTSD | Psychological Abuse |
| Baldry, 2003 | 145 | Italy | 37.8 (9.17) | F | Women shelter | CS | PMWI, SVAWS | Intrusion, anxiety/depression | Psychological abuse |
| Barchi, 2018 | 469 | Botswana | 31y | F | Community sample | CS | The DHS domestic violence module | Depression | Emotional violence |
| Basile, 2004 | 380 | US | 39.6 (12.38) | F | National sample (positive IPV response) | CS | CTS (physical)  Self-con: sexual, psychological, and stalking | PTSD | Psychological violence |
| Beach, 2004**^+^** | 166 | US | M: 41.2, W: 38.6 | F | Married couples | LS | CTS | Depression | Psychological aggression |
| Bebanic, 2017 | 19386 | Norway | 46 | F/M | Nationwide survey | CS | Overall | Depression, anxiety | Psychological violence |
| Beck, 2011 | 63 | US | 36.75 (11.62) | F | Women seeking treatment for mental health problems following IPV | CS | PMWI | PTSD | Psychological abuse |
| Becker, 2010 | 363 | US | 32.9 (5.2) | F | Mothers mixed sample (i.a.) | CS | CTS (physical/sexual) Psychological violence by 10-items: adapted from the CTS | PTSD | Psychological abuse |
| Beeble, 2009**^+^** | 160 | US | 32.1 (6.1) | F | Mixed community sample following legal involvement | LS | CTS, the index of psychological abuse | Depression | Psychological abuse |
| Beeble, 2010**^+^** | Same study as above (Beeble, 2009) | | | | | | | | |
| Blasco-Ross, 2010 | 91 | Spain | 45 | F | Women from IPV centres + a control group | LS | Overall physical, sexual, psychological | PTSD, depression, anxiety | Psychological violence |
| Bonomi, 2006**^+^** | 3429 | US | 45.3 | F | Women through health maintenance organization | CS | WEB + 5 items from the BRFSS | Depression | Non-physical IPV (fear, threats, power, control) |
| Brar, 2020**^+^** | 995 | Malawi (here) | 19.2 (2.5) | F | Data from the Girl Power study; young women | CS | CTS2, 5-items on controlling behaviour | Depression | Emotional abuse (and controlling behaviour) |
| Calvete, 2007a | 298 | Spain | 39.2 (11.8) | F | IPV victims – data from larger study | CS | CTS2, PAI | Depression | Psychological abuse |
| Calvete, 2008 | Same study as above | | | | |  | CTS2, PAI | Anxiety | Psychological abuse |
| Chandra, 2009 | 105 | India | 36 (9) | F | Mental health unit (non-psychotic) | CS | ISA | PTSD, depression | Psychological abuse |
| Chen, 2009 | 146 | US | 37.2y | F | Hispanic women from urban family medicine practice | CS | HITS, ISA -NP Controlling behaviours 7-items, WAST | Depression | Nonphysical abuse |
| Cheung, 2017 | 1198 | China | 42.7 (9.9) | F | Women from 13 community agencies and two shelters (without known abuse also included) | CS | PMWI, CTS2, CBS-R | Depression | Psychological maltreatment |
| Choi, 2020 | 3565 | Korea | 20 - >70 | F/M | Data KOWEPS; Nationally representative sample of households | LS | Overall for malicious or insulting, threatening, and violent behaviours | Depression | Verbal aggression |
| Chowdhary, 2008 | 1750 | India | 35.37 (6.6) | F | Married women (community) | LS | Overall for verbal, physical and sexual | Depression | Verbal violence |
| Coker, 2002a**^+^** | 1152 | US | 18->50 | F | Women who sought medical care | CS | ISA-P, WEB Abused Assessment Screen | Physical/mental health | Psychological violence |
| Coker, 2002b**^+^** | 13912 | US | 18-66 | F/M | National Violence Against Women Survey (NVAWS) | CS | CTS, National Women’s Study, Power & Control Scale | Depression, alcohol and drug abuse, chronic mental disease | Psychological abuse |
| Coker, 2005b | 554 | US | 18-66 | F/M | Data from the National Violence Against Women Survey (NVAWS); Same as Coker, 2002b | CS | CTS, the National Women's Study, Tjaden and Thoennes' measure of stalking, 7-item power and control subscales | PTSD | Power and control |
| Comechancha, 2017 | 661 | Portugal | 23.5 (4.5) | F/M | E-survey with university students | CS | PMWI, CTS2 | PTSD, depression, anxiety | Psychological violence |
| Cook, 2006 | 403 | US | 34.3 (8.03) | F | Female prison sample |  | CTS, Sexual Experience Scale, Conflict and Coercion dimensions: 13-items validating new scale | PTSD, depression | Psychological violence + coercion |
| Cowden, 2019 | 515 | South Africa | 29.5 (10.1) | F | Community sample | CS | PMWI | Depression, anxiety, stress | Psychological abuse |
| Dardis, 2018**^+^** | 101 | US | 49.4 (14.2) | F | Women veterans | LS | HARK, CTS2 | PTSD | Psychological violence |
| Dardis, 2019 | 318 | US | 19.02 (1.19) | F | College psychology students | CS | CTS2 | PTSD, depression | Psychological IPV |
| de Mendonca, 2017 | 390 | Brazil | 24->28 | F | Pregnant women | LS | Overall physical, psychological, and sexual | CMD | Psychological violence |
| deOliveira, 2017**^+^** | 456 | Brazil | <20- >35 | F | Sample from a maternity service | CS | CTS2 | PTSD | Psychological violence |
| del Rio, 2017 | 10171 | Spain | 16->60 | F | Nationally representative: the Spanish Survey on Violence Against Women | CS | Physical, sexual, emotional, economic, and controlling behaviour | Symptoms of poor health, e.g., sadness, insomnia, fatigue, sexual desire | Emotional violence (also assesses controlling behaviours separately) |
| DePrince, 2014 | 192 | US | 34.3 (11.0) | F | Police-reported IPV cases | CS | CTS-R | PTSD | Psychological aggression |
| Desmarais, 2014 | 100 | Canada | 32.5 (5.0) | F | Women within three months postpartum (add) | CS | CTS2 | PTSD, depression, anxiety, stress, OCD | Psychological aggression |
| Dichter, 2014 | 249 | US | 46.6 | F | Female veterans from medical centre (add) | CS | CTS2 | PTSD, depression/anxiety/bipolar, overall health, difficulty sleeping, problem drinking | Psychological violence |
| dos Santos, 2018 | 369 | Brazil | 20-59 | F | Women attending nursing consultations. | CS | CTS2 | Depressive/anxious moods, depressive thoughts, somatic symptoms, decreased vital energy | Psychological aggression |
| Duerksen, 2019 | 278 | Canada | 20.5 (1.9) | F/M | College psychology students | CS | CTS2, SES the SVQ, CARS. | PTSD, depression, stress, quality of life, alcohol | Psychological victimization |
| Dutton, 1999 |  |  |  |  |  |  |  |  |  |
| Ely, 2011 | 188 | US | 25 (6) | F | Women seeking abortion | CS | BAAS | Depression, perceived stress, aggression, suicide, self-esteem, alcohol abuse, drug use, sexual discord | Emotional abuse |
| Eshelman, 2012**^+^** | 499 | US | Most 19-20 | F | University (psychology) students | CS | SVAWS, PMWI, Injury Checklist | PTSD, depression, suicidality | Psychological abuse |
| Esie, 2019 | 3902 | Bangladesh | 24.4 (.11) | F | National representative longitudinal study | LS | CTS2 and he WHO standardized questionnaire on IPV | Depression | Psychological IPV |
| Eslami, 2017**^+^** | 4467 | Europe | 60-84 | F/M | Elder men and women in seven European countries | CS | CTS2 + the UK survey of abuse/neglect of older people | Depression, anxiety | Psychological abuse |
| Estafan, 2016 | 156 | US | 17-45+ | F | Community based collaborative program providing legal and case management services to victims of IPV | LS | Overall, physical, psychological, and sexual | Depression | Emotional abuse |
| Fahmy, 2008 | 500 | Egypt | 18-50 | F | Women attending health facilities | CS | Self-con Physical, psychological, social | Depression, anxiety | Psychological + social abuse |
| Fernández-Montalvo, 2017**^+^** | 180 | Spain | 37.5 (8.5) | F | Female substance abuse addicts entering treatment | CS | Not specified (physical, psychological, sexual) | Depression, anxiety, suicidal ideation, suicide attempts, hallucinations | Psychological abuse |
| Fisher, 2011**^+^** | 995 | US | 55-90 | F | Elder women contacted through primary care clinics | CS | Women’s Health and Relationship Survey (WHRS) | Depression or anxiety, poor health, chronic pain | Psychological abuse |
| Follingstad, 2012 | 200 | US | 45.3y | F | National sample of women in conflictual relationships (Web panel) | CS | MPAB, Measures on attributions regarding presence of psychological abuse | Depression, anxiety, somatization | Psychological abuse |
| Friborg, 2015**^+^** | 12762 | Norway | 57.5 (12.7) | F/M | Tromsø study; general population-based multi-purpose study (lifestyle-related diseases) | CS | Overall physical and threat | Mental health, muscular pain | Psychological violence |
| Garcia-Linares, 2004 | 111 | Spain | 41.7 (10.3) | F | IPV victims from help centres and non-exposed controls | CS | Overall physical, psychological, and sexual | PTSD, depression, anxiety | Psychological violence |
| Gibbs, 2018a | 935 | Afghanistan | 18-40+ | F | Women International (WfWI) intervention trial in Afghanistan; Married women | CS | WHO Multi-Country study | PTSD, depression, food insecurity, disability severity, life satisfaction, health rating, suicidal ideation | Emotional IPV |
| Gobin, 2013 | 425 | US | 34.5 (8.1) | F | Female victims exposed to physical IPV; 56.7% living in shelters | CS | CTS2 | PTSD | Psychological IPV |
| Gou, 2019**^+^** | 196 | Canada | F: 29.98 (5.49) M: 32.03 (5.51) | F/M | Couples pregnant with their first child | LS | CTS2, adapted-PMWI | Depression, alcohol | Coercive control |
| Grandin, 1998 | 1124 | Canada | Not reported | F/M | Community couples | CS | CTS2 | Depression, anxiety | Psychological abuse |
| Greene, 2018 | 308 |  | 26.1 (6.0) | F | Mothers from the MAPS Study; Oversampled based on IPV victimization | CS | CTS2 | PTSD | Psychological violence |
| Gucek, 2018 | 161 | Slovenia | 51.1 (16.8) | F/M | Follow-up on previous IPV study involving family doctors | CS | A domestic violence questionnaire previously used in Slovenian studies | Depression | Emotional violence |
| Haj-Yahia, 2000a | 1400 | Israel | 20.8 (2.9) | F | Random sample of engaged women; Arabic population | CS | CTS2 | Depression, anxiety, stress, self-esteem | Psychological aggression |
| Haj-Yahia, 2000b | 1334 | Palestine | 31.49 (9.10) | F | National Survey on Violence Against Women; random geographic distribution | CS | Questionnaire based on CTS2, PMWI, ISA with a total of 32 items (physical, psychological, sexual, and economic) | Depression, anxiety, self-esteem | Psychological abuse |
| Han, 2019 | 9217 | Korea | 19-60+ | F/M | KOWEPS; a nationally representative sample of married people | LS | CTS2 | Depression | Verbal aggression |
| Hansrod, 2015 | 169 | South Africa | 29.9 (7.3) | F | Convenience sample of HIV infected women + negative control | CS | Not specified; physical, psychological, and sexual abuse | PTSD, depression | Psychological aggression |
| Hassan, 2012 | 416 | Pakistan | 33 (9.5) | F | Married women living in Karachi city | CS | KDVSS-U | Depression, self-esteem, life satisfaction | Psychological abuse |
| Hazen, 2008**^+^** | 282 | US | 27.74 (7.12) | F | Latin women who had received community health care | CS | CTS2, PMWI-SF, LTE | Depression, anxiety, hostility, phobic anxiety, somatization, self-esteem | Psychological abuse (ver/emo and dom/iso) |
| Hedin, 1999 | 207 | Sweden | 29.5 (4.5) | F | Women from three antenatal clinics | CS | PMWI, SVAW | Anxiety, dissociation | Psychological abuse |
| Hegarty, 2004 | 1257 | Australia | 16-50 | F | Women recruited through general practitioners | CS | CAS | Depression | Emotional abuse/harassment |
| Hegarty, 2013 | 272 | Australia | 16-50 | F | Women who had attended urban and rural general practitioners in the last 12 months | CS | CAS | PTSD, depression, quality of life, general health, alcohol, somatization, etc | Emotional abuse/harassment |
| Hellemans, 2014**^+^** | 1472 | Belgium | W: 42.3 (14.4) M: 47.5 (14.9) | F/M | Subsample of a population-based survey on IPV | CS | CTS, MMEA,  2-item sexual violence. | Depression/anxiety, general health, stress, sleeping, alcohol, relationship quality, suicide | Psychological IPV |
| Henriques, 2015**^+^** | 456 | Brazil | <20 - >36 | F | Postnatal women attending a referral service for high-risk pregnancies | CS | THQ, CTS2 (physical and sexual) | PTSD | Psychological violence |
| Hicks, 2003**^+^** | 181 | US | 34 (12) | F | Chinese American women | CS | CTS2 + threats items | Depression | Threats |
| Hines, 2016 | 611 | US | 43.9 (9.2) | M | Help-seeking men following an episode of physical IPV victimization | CS | CTS2, PMWI (9-items controlling behaviours), the Actual LA Aggression scale | PTSD, depression, self-perceived health | Psychological aggression, controlling behaviours, legal/administrative aggression |
| Hines, 2011 | 822 | US | H-S 40.5 (9.0), Com 43.7 (10.9) | M | Mixed sample of IPV help-seeking men (N = 302) and a community sample (N = 520) | CS | CTS2, PMWI (7-items harm) | PTSD | Psychological violence and controlling behaviours |
| Holmes, 2020 | 398 | US | 19.7 (1.99) | F | College psychology students | CS | SVAW, PMWI, SBC, the modified electronic intrusion scale | PTSD, alcohol | Psychological victimization |
| Houry, 2006 | 569 | US | 34.2y | F | African American emergency department patients | CS | The George Washington University Universal Violence Prevention Screening Protocol | PTSD, depression | Emotional violence |
| Huang, 2001 | 140 | US | - | F/M | African American university students and faculty staff | CS | Overall physical and behavioural | Depression, Alcohol | Behavioural abuse |
| Huth-Bocks, 2013 | 120 | US | 26 (5.7) | F | Pregnant women in the third trimester | CS | CTS2 | PTSD | Psychological IPV |
| Ireland, 2017**^+^** | 381 | Australia | 34.8 (11.2) | LGBTI | LGBTI community (online survey) | CS | MMEA | Anxiety and hyperarousal, employment, intrusion | Hostile ignorance and control of communication; Social control suspicion, possessiveness: threats to possessions |
| Iverson, 2017**^+^** | 407 | US | 21-70 | F/M | Post 9/11 veterans | CS | CTS2 | PTSD, depression, health, occupation | Psychological IPV |
| Jaquier, 2015 | 143 | US | 38.1 (10.7) | F | Urban community (add); women exposed to > 1 act of physical IPV and used alcohol or drugs >1 in the last month | CS | PMWI, CTS2, SES | PTSD, anxiety, alcohol/drug | Psychological IPV |
| Jeter, 2014 | 232 | US | 18.3 (1.6) | F | Female graduate students (course credits) | CS | The Bully Victimization Scale, MPAB, CTS2 | PTSD | Psychological trauma by partner |
| Jina, 2012 | 1193 | South Africa | 18.2 (1.6) | F | Rural women in RCT on HIV behavioural (prevention) intervention; Baseline data | CS | Adapted-WHO violence against women instrument | Depression, distress, alcohol, drugs, suicidality | Emotional abuse |
| Jonas, 2014 | 7047 | England | >16 | F/M | National survey | CS | BCS | PTSD, CMD, alcohol, drugs, eating disorder, psychosis | Emotional abuse |
| Jones, 2005 | 172 | US | 27.7y | F | New mothers | CS | SOSPS, PWMI, SVAWS | PTSD, depression, anxiety, self-esteem | Psychological abuse |
| Katz, 1999 | 82 | US | 19 (1.15) | F | Undergraduate students; six weeks follow-up | LS | PMWI | PTSD | Psychological abuse |
| Karmaliani, 2009**^+^** | 1368 | Pakistan | <20->30 | F | Pregnant women | CS | Not specified | Depression and anxiety (joined) | Verbal abuse |
| Kastello, 2016**^+^** | 239 | US | 14-30+ | F | IPV exposed pregnant women | CS | CTS2 | PTSD | Psychological violence |
| Kelly, 2010b | 33 | US | 39.7 | F | Women from domestic service agency | CS | SVAWS, Appraisal of Violent Situations (women’s perceptions of abuse severity) | PTSD, depression | Psychological abuse (threats) |
| Kiene, 2017 | 325; 160 M and 165 F | Uganda | F: 32.2 (8.9)  M: 34.9 (10.59) | F/M | Data from a HIV risk reduction intervention | CS | Adapted-WHO violence against women instrumen | Depression, alcohol | Emotional IPV |
| Kinyanda, 2016 | 1110 | Uganda | 15-65+ | F/M | Data from the Uganda AIDS. | CS | An 11-item questionnaire based on the Intimate Partner Violence Assessment Questionnaire | Depression, alcohol, suicide | Psychological IPV |
| Koopman, 2007 | 57 | US | 35.5 (9.4) | F | Women exposed to IPV (add). | CS | The Abusive behaviour Inventory Partner Form | Depression, bodily pain | Psychological abuse |
| Kramer, 2004**^+^** | 1268 | US | 18-44 | F | Females from emergency departments and primary care clinics | CS | Abuse Assessment Screen | Depression, health, suicide | Emotional abuse |
| Lacey, 2013 | 8000 | US | 18-54 | F | NVAWS; National IPV Survey | CS | Modified-CTS, 8-items on stalking, 1-item threat | Depression, alcohol, substance, health | Psychological abuse |
| Lawrence, 2009**^+^** | 103 couples | US | F: 25.0 (4.3)  M: 26.4 (4.7) | F | Couples | LS | MMEA, CTS2 | Depression, anxiety | Psychological victimization |
| Levine, 2016 | 51 | Canada | 37 | F | Homeless women (shelter) with physical IPV experience | CS | CTS2, CBS-R | PTSD, depression | Coercive control |
| Lilly, 2009**^+^** | 132 | US | 33.5 (5.8) | F | Subsample of mothers from a larger study (witnessing IPV; add) | CS | CTS2, Severity of Violence Against Women Scale | PTSD | Psychological violence |
| Lilly, 2010 | 97 | US | 33.2 (8.7) | F | Women with children recruited from shelters and adds | CS | CTS2 | PTSD | Psychological aggression |
| Longares, 2016 | 357 | Spain | 29.1 (9.9) | F/M | Lesbian and gay men (most single); online study | CS | EAPA-P | Depression | Psychological abuse |
| Longares, 2018 | 372 | Spain | 31.03 (9.77) | F/M | Individuals with same-sex sexual orientation | CS | 1-item for self-identification of psychological abuse, EAPA-P | Depression, anxiety | Psychological abuse |
| Lovestad, 2017 | 573 | Sweden | 42.7 (13.01) | F | Random sample of Swedish women | CS | VAWI (The WHO Violence Against Women Instrument (physical, sexual), the Controlling Behaviours Scale (isolating control subscale) | Depression | Controlling behaviour (Isolating control) |
| Marshall, 1999**^+^** | 834 | US | 32.8 | F | The HOW project a longitudinal study with recruitment by advertisement and referral | LS | SVAWS, Men's Psychological-Harm and Abuse in Relationships Measure-Overt scales (MP-HARM-O) and -Subtle | Depression, self-esteem, stress, health quality, emotional distress, rumination | Psychological abuse |
| Martin, 2006 | 95 | US | 26.5 (6.4) | F | Women recruited from prenatal care clinics | CS | CTS2 | Depression | Psychological aggression |
| Matseke | 673 | South Africa | 28.4 (5.7) | F | Baseline data from the Protect Your Family | CS | CTS2 | Depression, alcohol | Psychological IPV |
| Matud, 2005 | 480 | Spain | 37.2 (10.1) | F | Abused women and control group | CS | PPAS | Depression, anxiety, somatic, social | Psychological abuse |
| McMahon, 2011 | 3961 | US | 16->30 | F | Pregnant women; the Fragile Families and Child Wellbeing Study | LS | Overall physical and psychological | Depression, health | Emotional abuse |
| Mechanic, 2008 | 413 | US | 34.5 (8.1) | F | Women from a community battered women’s program | CS | PMWI, CTS2, SBC | PTSD, depression | Psychological abuse |
| Mechanic, 2000 | 31 | US | 35 (7.9) | F | Acutely battered women | CS | PMWI, CTS2, SBC | PTSD, depression | Psychological abuse |
| Meekers, 2013**^+^** | 10119 | Bolivia | 15-49 | F | Data from the 2008 Bolivia Demographic and Health Survey; (married or women in union) | CS | Modified-CTS2 | Depression, anxiety, psychosis | Psychological abuse |
| Melander, 2020 | 540 | US | 19.5 (1.6) | F/M | College students | CS | CTS2, Cyber-IPV by self-con | Depression, substance use, anger, antisocial | Psychological abuse |
| Miller, 2017 | 4769 | US | 46.7 (13.47) | F/M | Data from the BRFSS (nationally representative); Comparing heterosexuals  and LGB exposed to IPV | CS | Over physical, sexual, threats | Depression, anxiety, sleep quality | Verbal threats of physical abuse |
| Miller-Graft, 2017 | 101 | US | 26 (5.7) | F | Pregnant women | CS | CTS2 | PTSD, depression, sleep | Psychological IPV |
| Mills, 2018 | 128 | US | 39.5 (11.6) | F | Community sample of African American women who experienced abuse | CS | PAS | PTSD, depression, anxiety | Nonphysical abuse |
| Mittal, 2018 | 175 | US | 36 (10.7) | F | Data from an RCT on integrated HIV-IPV prevention intervention for abused women | CS | ABI, WEB | Depression, self-esteem | Psychological abuse |
| Montgomery, 2015 | 2099 | US | 18->34; median 29 | F | Baseline data from the Women’s HIV SeroIncidence Study | CS | Overall physical, sexual, psychological | PTSD, depression, substance abuse | Emotional abuse |
| Mouton, 2010 | 93676 | US | 65 (9) | F | Data from the WHI Study; Post-menopausal women | LS | Overall physical and verbal | Depression, mental health | Verbal abuse |
| Mugoya, 2020 | 247 | Kenya | 34.5 (8) | F/M | Caregivers of children with disabilities | CS | CTS2 | Depression | Psychological aggression |
| Mugoya, 2020 | 664 | US | 38.9 (12.9) | F | Adult African American female caregivers | CS | CTS2 | Depression | Psychological aggression |
| Nathanson, 2012 | 202 | US | 32.9 (9.5) | F | The Women’s Health Study: Current or recent relationship (through add) | CS | CTS2 | PTSD, depression, alcohol/drug | Psychological aggression |
| Nixon, 2004**^+^** | 142 | US | 34.4 (8.2) | F | Women exposed to physical abuse from IPV help agencies | CS | CTS2 (extra items), BASIS-32, SAEQ (the Sexual Abuse Expo-sure Questionnaire, AE-III-PP | PTSD, depression | Psychological aggression |
| Nedd, 2001 | 128 | US | 30.93 (8.13) | F | African American women who had problems, including violence with their intimate partners | CS | ISA | Depression, stress | Emotional abuse |
| Nnawulezi, 2019 | 228 | US | 34.2 (10.1) | F | Archival records from an abuser intervention (partners) | CS | CTS2, MMEA | PTSD | Psychological aggression + psychological, emotional, and verbal abuse |
| Nurius, 2003 | 448 | US | 32 (9.5) | F | Female IPV victims reporting to police or filing a protection order | LS | CTS2, WEB | Depression, physical health | Psychological abuse |
| Overstreet, 2015 | 186 | US | 35.9 (10.5) | F | Community women currently experiencing IPV | CS | PMWI, CTS2, SES | PTSD | Psychological IPV |
| Pantalone, 2012 | 168 | US | 44.0 (8.4) | M | Men from outpatient public university-affiliated HIV clinics | CS | CTS2 | Depression, anxiety, suicide, substance, health | Psychological abuse |
| Panuzio, 2007 | 52 | US | 35.0 (7.4) | F | Female partner of male clients of treatment program for perpetrators | CS | CTS, MMEA | PTSD | Psychological aggression |
| Peltzer, 2017 | 207 | Thailand | 26.8 (9.3) | F | Women from antenatal care or general outpatient clinics; Victims of physical or sexual IPV | CS | SVAWS, DA | Depression, suicide | Psychological violence |
| Pickover, 2017 | 284 | US | 37.8 (12.1) | F | Women assessed by a mental health research clinic for female survivors of IPV (add) | CS | CTS2, PMWI, CPQ (The Communication Patterns Questionnaire) | PTSD, anxiety | Psychological abuse |
| Pico-Alfonso, 2005**^+^** | 127 | Spain | 44.2 (11.7) | F | Female IPV victims from help centres (+ non-exposed control group) | CS | Overall self-constructed for physical, sexual, and psychological | PTSD | Psychological violence |
| Pico-Alfonso, 2006 | 182 | Spain | 44.2 (11.7) | F | Female IPV victims from help centres (+ non-exposed control group) | CS | Self-con for physical (1-QN), sexual (10-QN) and psychological (1-QN) | PTSD, depression, anxiety | Psychologically abused |
| Pantalone, 2012**^+^** | 168 | US | 44.0 (8.4) | M | Men from outpatient public university-affiliated HIV clinics | CS | CTS2 | Depression, anxiety, suicide, substance, health | Psychological abuse |
| Postmus, 2012 | 2305 | US | 25.8 (6.1) | F | The FFCWS; study on parents and the well-being of their children (here mothers) | LS | Self-con items on physical, sexual, psychological, and economic | Depression | Economic and psychological abuse |
| Prospero, 2010**^+^** | 370 | US | 21.9 (4.2) | M | University students | CS | CTS2, Additional self-con on sexual IPV | Depression, anxiety, hostility, somatic | Verbal/psychological abuse |
| Ratner, 1993 | 406 | Canada | 39.4 (14.3) | F | Married or cohabiting women | CS | CTS | Depression, anxiety, somatic, alcohol | Psychological abuse |
| Rauer, 2010 | 241 couples | US | F: 33.4 (6.0), M: 36.4 (6.6) | F/M | Semi-rural couples with child(ren) | LS | SOPAS, SVAMS and SVAWS | Depression, anxiety, sleep | Psychological abuse |
| Rauer, 2012 | 215 | US | F: 33.4 (6.0), M: 36.4 (6.6) | F/M | Semi-rural couples with child(ren) | LS | CTS2 | Depression, sleep | Psychological abuse |
| Reid, 2008**^+^** | 445 | US | 53.8 (15.9) | M | Men recruited from a non-profit health-care system | CS | Questions from the U.S. Behavioural Risk Factor Surveillance System (BRFSS) survey: non-physical (threats, controlling), physical and sexual | Depression, Health | Nonphysical IPV |
| Rees, 2016 | 1672 | Timor-Leste | 20->35 | F | Pregnant women in the second trimester | CS | The WHO Multi-Country Study on Women’s Health and Domestic Violence measure | PTSD, depression, psychological distress | Psychological abuse |
| Reich, 2015 | 79 | US | 36.1 (11.7) | F | Women with a history of IPV who sought help for mental help concerns | CS | CTS2 | PTSD, self-esteem | Psychological abuse |
| Renner, 2009 | 1153 | US | 31.9 (7.7) | F | Lower-income women; Data IFS | CS | Self-con adapted from: Massachusetts study of women on welfare, CTS2, WES and WEB | Depression, health, substance | Psychological IPV |
| Rogers, 2014**^+^** | 361 | US | 45.9 (12.02) | F | Women in a conflictual or problematic relationship (online panel) | CS | Self-con by author (physical/sexual), MPAB, PH (Global Perceived Harm), WEB, OR (The Over-Reporting Style Questionnaire) | Depression, anxiety, functioning, suicide | Psychological abuse |
| Roh, 2016 | 233 | US | 60.7 | F/M | Indigenous older adults | CS | CTS2 | Depression | Psychological aggression |
| Rurangirwa, 2018 | 921 | Rwanda | 15-47 | F | Women who had given birth < 13 months before | CS | The Women’s Health and Life Experiences Questionnaire (WHO) | PTSD, Depression, anxiety, suicide | Psychological IPV and controlling behaviour |
| Sabina, 2008**^+^** | 4533 | US | 21.65 | F/M | Data from the IDVS; Dating violence among college students | CS | CTS2 | PTSD, depression | Psychological aggression |
| Sabri, 2013a**^+^** | 543 | US and St. Croix and St. Thomas in the U.S. Virgin Islands | 29.3 | F | Recruited from primary care, prenatal or family planning clinics (reporting IPV) | CS | WEB, SVAWS, DA | PTSD, depression, substance use | Psychological abuse |
| Sabri, 2013b | 431 | US and St. Croix and St. Thomas in the U.S. Virgin Islands | 28.2 (8.2) | F | Same study as described above, but only | CS | WEB, SVAWS, DA | PTSD, depression, psychological distress | Psychological IPV |
| Sackett, 1999 | 60 | US | 34.7 (9.1) | F | Women seeking help from domestic violence agencies; all physically abused | CS | Profiles of psychological abuse (developed for this study | Depression, self-esteem | Psychological abuse |
| Salwen, 2015**^+^** | 586 | US | 1) 35.1 (5.0); 2) 19.8 (1.7) | F | Combined samples;  1) Women living with partners and children; and 2) undergrad students | CS | CTS2 | Depression | Psychological aggression |
| Sargent, 2016 | 332 | US | 18.3 (.8) | F/M | First-year college students | CS | PCAQ, CADRI | Depression | Psychological abuse and cyber-abuse |
| Sauber, 2017 | 147 | US | 37.6 (11.6) | F | Low-income women exposed to domestic abuse | CS | ABI, SEA-12 | PTSD, depression | Psychological abuse |
| Shamu, 2016 | 842 | Zimbabwe | 15-49 | F/M | Postnatal women | CS | The WHO questionnaire for measuring violence against women | Depression, suicide | Emotional violence |
| Shen, 2014 | 1018 | Taiwan | 21 (1.7) | F/M | Unmarried men and women; College students | CS | The Dating Violence Scale (by author) | PTSD | Psychological violence |
| Shen, 2019 | 726 | US | 18-20 | F | The Relationship Dynamic and Social Life study; a population-representative sample from one Michigan count | CS | Overall physical and psychological | Depression, stress, self-esteem | Psychological violence |
| Shorey, 2011**^+^** | 967 | US | 19.7 (2.9) | F/M | Undergraduate college students | CS | CTS2 | Depression, anxiety, guilt, and shame | Psychological aggression |
| Shorey, 2012 | 184 | US | 19.4 (1.6) | M | College students | CS | CTS2, PMWI | PTSD | Psychological victimization |
| Signorelli, 2012 | 31 | Italy | - | F | Women recruited by an anti-violence centre (abstract only) | CS | CTS2 | Depression | Psychological violence |
| Simonelli, 1998 | 70 | US | 21.4 (4.8) | M | Undergraduate psychology students | CS | PMI, CTS | Depression, anxiety | Psychological maltreatment and verbal aggression |
| Smagur, 2018 | 206 | US | 25.4 (5.0) | F | Data from a larger longitudinal study; women in their third trimester; Roughly half had experienced IPV during pregnancy | CS | SVAWS, CTS2 | Depression | Emotional IPV |
| Sotskova, 2013 | 167 | Canada | W: 31 (5.0), M: 34 (4.9), | F/M | Couples one year after the birth of their first child | CS | CTS2 | PTSD, depression, alcohol | Psychological IPV |
| Street, 2001 | 63 | US | 32 | F | Women seeking help from 23 battered women shelter | CS | CTS2, PMWI | PTSD, depression, shame and guilt | Psychological abuse |
| Stylianou, 2018 | 457 | US | 36 (9.2) | F | Women recruited from 14 domestic violence agencies | CS | ABI-R, SEA | PTSD | Psychological abuse, economic abuse |
| Sullivan, 2013 | 354 | US | 36.7 (9.01) | F | Women in bidirectional (physical) violent relationships | CS | CTS2, SES, PMW | PTSD, depression, anxiety | Psychological aggression |
| Sullivan, 2009 | 212 | US | 36.6 (10.5) | F | Women in (physical) violent relationships | CS | CTS2, The Past Abusive Relationships | PTSD, alcohol, and drug abuse | Psychological IPV |
| Tadegge, 2008 | 515 | Ethiopia | 33.1 (10.9) | F | Ever partnered women: systematically random selected households | CS | The Amharic version of the structured World Health Organization (WHO) domestic violence questionnaire | Depression, mental distress, and suicide | Emotional abuse |
| Tang, 1997 | 59 | Hong Kong | pprox.. 37 | F | Comparing three groups; shelter, help seeking for marital problems and women from intact relationships | CS | CTS2 | Depression, anxiety, distress | Verbal abuse |
| Tang, 1998 | 72 | Hong Kong | 35-37 | F | Abused group (shelter) and non-abused from the local community | CS | CTS2, ISA | Depression, anxiety, general distress | Nonphysical and verbal abuse |
| Terrazas-Carrillo, 2016**^+^** | 13053 | Mexico | 47.4 (13.8) | F | National survey: women who identified as married or cohabiting | CS | Scale with 29-items measuring physical, emotional, and sexual IPV (joined scale), 12-items measuring coercive control; not specified | Depression | Coercive control (emotional abuse included in pooled IPV score) |
| Theran, 2006 | 398 | US | 34.2 | F | Newspaper ad; abused and non-abused women of both low- and middle income | CS | CTS2, IPA | Depression, stress | Emotional abuse |
| Thompson, 1999 | 204 | US | 1) 31.4; 2) 38.3 | F | Sample from Grady Health System; 1) women with nonfatal suicide attempt and 2) non-abused/non-suicidal controls | CS | ISA | PTSD, suicide | Nonphysical abuse |
| Thompson, 2000 | 138 | US | 31.3 (9.6) | F | African American women recruited from medical walk-in clinics | CS | ISA | Depression, anxiety, psychological distress | Non-physical abuse |
| Tiwari, 2013 | 308 | Hong Kong | 40.8 (8.8) | F | Women recruited from women shelters | CS | C-AAS, CTS2 | PTSD, depression, chronic pain | Psychological abuse |
| Torres, 2000 | 124 | US | 32.3 (8.9) | F | Women shelters or outreach shelter offices | CS | CTS2 | PTSD, depression, anxiety | Nonphysical abuse |
| Torres, 2019 | 46 | US | 41.5 (11.9) | F | Victims from sexual assault and domestic violence centres (one identified as male) | CS | MMEA, CTS2 | PTSD | Emotional abuse |
| Tung, 2019 | 200 | US | 19.8 (1.3) | F | The Pittsburgh Girls Study; from low-income neighbourhoods | LS | ACE, CTS2 | PTSD, emotional distress | Emotional IPV |
| Tuthill, 2019**^+^** | 211 couples (422 individuals) | Malawi | 40.5 (10.2) | F/M | HIV-positive individuals and their partners | CS | Questions from the WHO domestic violence module | Depression | Emotional abuse |
| Tyson, 2007 | 92 | US | 31.4 (8.04) | F | From battered women shelters | CS | PAS | PTSD | Psychological abuse |
| Umubyeyi, 2014 | 917 | Rwanda | 20-35 | F/M | Population-based study; a representative sample | CS | Women’s Health and Life experiences questionnaire developed by WHO | PTSD, depression, anxiety, suicide | Psychological violence |
| Vargas, 2015 | 136 | US/Mexico | 31.8 (5.3) | F | Mothers living as Mexican immigrants in Texas and in Mexico; recruitment through kindergarten enrolment | CS | CTS2 | Depression | Psychological abuse |
| Varma, 2007 | 203 | India | 23 | F | Antenatal outpatient clinic; pregnant women | CS | ISA, SES | Depression, somatic | Psychological IPV |
| Wangel, 2016 | 1003 | Sweden | <25 - > 35 | F | Pregnant women) in the gestational weeks 27–30; Swedish data (Biden study) | CS | NorAQ | PTSD, depression | Emotional abuse |
| Weiss, 2015 | 197 | US | 36.97 (10.43) | F | African American women reporting >1 episode of physical IPV | CS | CTS2, PMWI, SES | PTSD, self-harm | Psychological IPV |
| Wijma, 2007 | 4150 | Sweden | 37 (12.2) | F | Women randomized from the population register in the Swedish country | CS | ASI | Depression, anxiety, somatic, sleep | Psychological IPV |
| Wolford-Clevenger, 2016 | 502 | US | 20.23 (4.41) | F/M | College (psychology) students | CS | CAS | Depression, suicidal ideation | Emotional abuse |
| Wolford-Clevenger, 2017a | 208 | US | 19.6 (11.09) | F/M | Couples recruited from introductory psychology courses | CS | CTS2, MMEA | Suicide | Emotional abuse |
| Woods, 2000 | 160 | US | 31.85; 38.13; 31.65 | F | Comparative design with three groups; Abused, post-abused and non-abused | CS | ISA, DA | PTSD | Emotional abuse |
| Woods, 2010 | 157 | US | 33.7 (9.5) | F | Women from three crisis IPV shelters and community agencies | CS | SVAWS, ISA-NP | PTSD, depression, stress, sleep | Emotional abuse and threats |
| Woods, 2008 | Same study as Woods (2010); new outcome | | | | | | | PTSD | Emotional abuse and threats |
| Wuest, 2010 | 309 | Canada | 39.4 (9.8) | F | Community women who ended abusive relationship | CS | ISA | PTSD, depression, pain | Psychological IPV |
| Yoshihama, 2002**^+^** | 211 | US | 37.2 (10.2) | F | Women of Japanese descent | CS | CTS2 (physical), emotional from various scale | PTSD | Emotional abuse |
| Yuan, 2019 | 2987 | China | 36.5 (9.5) | F | Community women | CS | Items from CTS2 and CAS | Depression | Psychological violence |
| Zacarias, 2012 | 1442 | Mozambique | 28.7 (8.1) | F | Women visiting the Central Hospital for partner abuse | CS | CTS2, CBS-R | Depression, anxiety, somatic | Psychological violence and controlling behaviours |

***Note.*** *G = gender; **^+^** = only for qualitative synthesis (e.g., Ali, 1999**^+^**).
