# Supplementary material for: The psychological subtype of intimate partner violence and its effect on mental health: a systematic review with meta-analyses
Source: Syst Rev. 2022 Aug 10;11:163. doi: 10.1186/s13643-022-02025-z (PMC9364557; doi:10.1186/s13643-022-02025-z)
Supplement: Supplementary file 2 — Additional file 2. PTSD Subtype Analyses – Female Victimization [file 13643_2022_2025_MOESM2_ESM.docx]

| **PTSD Subtype Analyses – Female Victimization** | | | | | | | | | | | |
| --- | --- | --- | --- | --- | --- | --- | --- | --- | --- | --- | --- |
| **Hedges g** | | | | | | **Odds Ratio** | | | | | |
|  | ***k*** | **Effect** | **95% CI** | **I^2^** | **τ^2^** |  | ***k*** | **OR** | **95% CI** | **I^2^** | **τ^2^** |
| **Scale measure** | | | | | | | | | | | |
| ***1. CTS2*** | 18 | 0.69 | [0.46: 0.92] | 92% | 0.2231, p < .01 | ***1. Valid IPV Scale*** | 8 | 1.81 | [1.18; 2.78] | 83% | 0.2293, p < .01 |
| ***2. PMWI*** | 8 | 1.02 | [0.73; 1.30] | 86% | 0.1410, p < .01 | ***2. WHO etc.*** | 3 | 3.06 | [0.98; 9.53] | 85% | 0.8315, p < .01 |
| ***3. ISA-NP*** | 2 | 1.11 | [-0.03; 2.25] | 94% | 0.6349, p < .01 | ***3. National item*** | 2 | 2.60 | [0.92; 7.31] | 83% | 0.4732, p < .02 |
| ***4. MMEA*** | 5 | 1.20 | [0.67; 1.73] | 89% | 0.3118, p < .01 |  |  |  |  |  |  |
| ***5. Other valid scale*** | 9 | 0.88 | [0.66; 1.09] | 68% | 0.0673, p < .01 |  |  |  |  |  |  |
| ***6. Other less known*** | 3 | 1.28 | [1.10; 1.47] | 0% | 0.0,  p = .39 |  |  |  |  |  |  |
|  | ***Residual heterogeneity: 93%, p < .01*** | | | | |  | ***Residual heterogeneity: 79%, p < .01*** | | | | |
| **Population** | | | | | | | | | | | |
| ***1. General*** | 2 | 0.17 | [-0.36; 0.71] | 87% | 0.1306, p < .01 | ***1. General*** | 3 | 2.39 | [1.61; 3.54] | 5% | 0.0059, p = .35 |
| ***2. Youth/ college*** | 3 | 0.68 | [0.55; 0.81] | 0% | 0.0,  p = .75 | ***2. Youth/ college*** | 0 | – | – | – | – |
| ***3. Clinical*** | 9 | 0.88 | [0.54; 1.21] | 89% | 0.2316, p < .01 | ***3. Clinical*** | 10 | 2.22 | [1.24; 3.99] | 96% | 0.7064, p < .01 |
| ***4. IPV*** | 31 | 0.98 | [0.82; 1.14] | 87% | 0.1687, p < .01 | ***4. IPV*** | 0 | – | – | – | – |
|  | ***Residual heterogeneity: 87%, p < .01*** | | | | |  | ***Residual heterogeneity: 95%, p < .01*** | | | | |
| **Culture** | | | | | | | | | | | |
| ***1. EU*** | 3 | 1.14 | [0.43; 1.85] | 87% | 0.3410, p < .01 | ***1. EU*** | 2 | 1.68 | [0.68; 4.13] | 6% | 0.0567, p = .30 |
| ***2. US/AU*** | 39 | 0.83 | [0.71; 0.96] | 85% | 0.1303. p < .01 | ***2. US/AU*** | 7 | 2.36 | [1.17; 4.78] | 97% | 0.7658, p < .01 |
| ***3. South America*** | 0 | – | – | – | – | ***3. South America*** | 0 | – | – | – | – |
| ***4. Africa*** | 0 | – | – | – | – | ***4. Africa*** | 4 | 2.09 | [0.97; 4.49] | 84% | 0.5113, p < .01 |
| ***5. Asia/ Middle East*** | 3 | 1.55 | [0.57; 2.52] | 97% | 0.7179, p < .01 | ***5. Asia/ Middle East*** | 0 | – | – | – | – |
|  | ***Residual heterogeneity: 87%, p < .01*** | | | | |  | ***Residual heterogeneity: 95%, p < .01*** | | | | |
| **Subtype psychological violence** | | | | | | | | | | | |
| ***1. Overall*** | 41 | 0.89 | [0.75; 1.04] | 89% | 0.1849, p < .01 |  |  |  |  |  |  |
| ***2. Threats*** | 1 | 0.29 | [-0.40; 0.97] | - | - |  |  |  |  |  |  |
| ***3. Control*** | 3 | 1.17 | [0.99; 1.34] | 0% | 0.0,  p = .46 |  |  |  |  |  |  |
|  | ***Residual heterogeneity: 88%, p < .01*** | | | | |  |  |  |  |  |  |
| **Study quality** | | | | | | | | | | | |
| ***1. Weak*** | 33 | 0.94 | [0.81; 1.08] | 83% | 0.1255, p < .01 | ***1. Weak*** | 2 | 2.60 | [1.24; 5.45] | 0% | 0.0,  p = .30 |
| ***2. Moderate*** | 8 | 0.90 | [0.46; 1.34] | 93% | 0.3740, p < .01 | ***2.Moderate*** | 10 | 2.08 | [1.19; 3.64] | 96% | 0.6661, p < .01 |
| ***3. Strong*** | 4 | 0.52 | [-0.06; 1.11] | 95% | 0.3332, p < .01 | ***3. Strong*** | 1 | 3.10 | [1.50; 6.41] | – | – |
|  | ***Residual heterogeneity: 88%, p < .01*** | | | | |  | ***Residual heterogeneity: 95%, p < .01*** | | | | |
