# Supplementary material for: The psychological subtype of intimate partner violence and its effect on mental health: a systematic review with meta-analyses
Source: Syst Rev. 2022 Aug 10;11:163. doi: 10.1186/s13643-022-02025-z (PMC9364557; doi:10.1186/s13643-022-02025-z)
Supplement: Supplementary file 3 — Additional file 3. Depression Subtype Analyses – Female Victimization [file 13643_2022_2025_MOESM3_ESM.docx]

| **Depression Subtype Analyses – Female Victimization** | | | | | | | | | | | |
| --- | --- | --- | --- | --- | --- | --- | --- | --- | --- | --- | --- |
| **Hedges g** | | | | | | **Odds Ratio** | | | | | |
|  | ***k*** | **Effect** | **95% CI** | **I^2^** | **τ^2^** |  | ***k*** | **OR** | **95% CI** | **I^2^** | **τ^2^** |
| **Scale measure** | | | | | | | | | | | |
| ***1. CTS2*** | *18* | 0.52 | [0.38; 0.66] | 82% | 0.0648, p < .01 | ***1. Valid IPV Scale*** | 11 | 1.70 | [1.23; 2.34] | 89% | 0.2236, p < .01 |
| ***2. ISA-NP*** | *7* | 0.70 | [0.49; 0.90] | 57% | 0.0383, p = .03 | ***2. WHO etc.*** | 8 | 2.43 | [1.57; 3.76] | 97% | 0.7626, p < .01 |
| ***3. PMWI*** | *5* | 1.07 | [0.58; 1.57] | 96% | 0.2928, p < .01 | ***3. National item*** | 12 | 2.27 | [1.27; 4.05] | 98% | 1.0011, p < .01 |
| ***4. Other known IPV*** | *17* | 0.78 | [0.57; 0.99] | 90% | 0.1638, p < .01 |  |  |  |  |  |  |
| ***5. Other unknown scale*** | *10* | 0.55 | [0.39; 0.71] | 92% | 0.0410, p < .01 |  |  |  |  |  |  |
|  | ***Residual heterogeneity: 89%, p < .01*** | | | | |  | ***Residual heterogeneity: 97%, p < .01*** | | | | |
| **Population** | | | | | | | | | | | |
| ***1. General*** | 16 | 0.65 | [0.48; 0.82] | 97% | 0.1050, p < .01 | ***1. General*** | 14 | 2.13 | [1.23; 3.70] | 98% | 0.0451, p < .01 |
| ***2. Youth/ college*** | 2 | 0.51 | [0.32; 0.70] | 95% | 0.0,  p = .77 | ***2. Youth/ college*** | 1 | 2.25 | [1.49; 3.40] | – | – |
| ***3. Clinical*** | 9 | 0.68 | [0.54; 0.81] | 28% | 0.0115, p = .20 | ***3. Clinical*** | 14 | 2.08 | [1.55; 2.79] | 85% | 0.2434, p < .01 |
| ***4. IPV*** | 30 | 0.75 | [0.56; 0.93] | 92% | 0.2212, p < .01 | ***4. IPV*** | 2 | 1.85 | [0.61; 5.62] | – | – |
|  | ***Residual heterogeneity: 94%, p < .01*** | | | | |  | ***Residual heterogeneity: 98%, p < .01*** | | | | |
| **Culture** | | | | | | | | | | | |
| ***1. EU*** | 7 | 0.84 | [0.53; 1.16] | 83% | 0.1359, p < .01 | ***1. EU*** | 4 | 2.92 | [1.99; 4.29] | 77% | 0.1173, p < .01 |
| ***2. US/AU*** | 37 | 0.59 | [0.49; 0.69] | 90% | 0.0676, p < .01 | ***2. US/AU*** | 11 | 1.93 | [0.92; 4.05] | 98% | 0.4777, p < .01 |
| ***3. South America*** | 0 | – | – | – | – | ***3. South America*** | 1 | 3.11 | [1.93; 5.01] | – | – |
| ***4. Africa*** | 2 | 0.66 | [-0.02; 1.34] | 94% | 0.2345, p < .01 | ***4. Africa*** | 11 | 1.95 | [1.36; 2.80] | 92% | 0.3092, p < .01 |
| ***5. Asia/ Middle East*** | 11 | 0.94 | [0.53; 1.35] | 98% | 0.4617, p < .01 | ***5. Asia/ Middle East*** | 4 | 1.94 | [1.18; 3.18] | 94% | 0.2250, p < .01 |
|  | ***Residual heterogeneity: 94%, p < .01*** | | | | |  | ***Residual heterogeneity: 96%, p < .01*** | | | | |
| **Subtype psychological violence** | | | | | | | | | | | |
| ***1. Overall*** | 52 | 0.70 | [0.58; 0.82] | 96% | 0.1618, p < .01 |  |  |  |  |  |  |
| ***2. Threats*** | 2 | 0.95 | [0.68; 1.22] | 0% | 0.1600, p < .01 |  |  |  |  |  |  |
| ***3. Control*** | 3 | 0.50 | [0.27; 0.73] | 0% | 0.0,  p = .65 |  |  |  |  |  |  |
|  | ***Residual heterogeneity: 95%, p < .01*** | | | | |  |  |  |  |  |  |
| **Study quality** | | | | | | | | | | | |
| ***1. Weak*** | 33 | 0.75 | [0.60; 0.89] | 84% | 0.1358, p < .01 | ***1. Weak*** | 6 | 3.00 | [1.92; 4.66] | 88% | 0.2500, p < .01 |
| ***2. Moderate*** | 21 | 0.63 | [0.45; 0.81] | 97% | 0.1465, p < .01 | ***2.Moderate*** | 18 | 2.22 | [1.63; 3.02] | 95% | 0.3722, p < .01 |
| ***3. Strong*** | 3 | 0.65 | [0.21; 1.08] | 92% | 0.1339, p < .01 | ***3. Strong*** | 7 | 1.25 | [0.56; 2.81] | 98% | 1.1537, p < .01 |
|  | ***Residual heterogeneity: 94%, p < .01*** | | | | |  | ***Residual heterogeneity: 94%, p < .01*** | | | | |
