# Supplementary material for: The psychological subtype of intimate partner violence and its effect on mental health: a systematic review with meta-analyses
Source: Syst Rev. 2022 Aug 10;11:163. doi: 10.1186/s13643-022-02025-z (PMC9364557; doi:10.1186/s13643-022-02025-z)
Supplement: Supplementary file 4 — Additional file 4. Depression Subtype Analyses – Male Victimization [file 13643_2022_2025_MOESM4_ESM.docx]

| **Depression Subtype Analyses – Male Victimization** | | | | | | | | | | | |
| --- | --- | --- | --- | --- | --- | --- | --- | --- | --- | --- | --- |
| **Hedges g** | | | | | | **Odds Ratio** | | | | | |
|  | ***k*** | **Effect** | **95% CI** | **I^2^** | **τ^2^** |  | ***k*** | **OR** | **95% CI** | **I^2^** | **τ^2^** |
| **Scale measure** | | | | | | | | | | | |
| ***1. CTS2*** | 6 | 0.43 | [.10; 0.76] | 89% | .1429, p < .01 | ***1. Valid IPV Scale*** | 3 | 1.06 | [0.68‚ 1.65] | 58% | 0.0850, p = .09 |
| ***2. PMWI*** |  | – | – | – | – | ***2. WHO etc.*** | 2 | 4.24 | [2.32; 7.74] | 0% | 0.0,  p = .52 |
| ***3. ISA-NP*** |  | – | – | – | – | ***3. National item*** | 1 | 11.68 | [7.02; 19.44] | – | – |
| ***4. MMEA*** | 1 | 0.19 | [-0.53: 0.91] | – | – |  |  |  |  |  |  |
| ***5. Other valid scale*** | 1 | 0.56 | [0.30: 0.82] | – | – |  |  |  |  |  |  |
| ***6. Other less known*** |  | – | – | – | – |  |  |  |  |  |  |
|  | ***Residual heterogeneity: 89%, p < .01*** | | | | |  | ***Residual heterogeneity: %, p < .0*** | | | | |
| **Population** | | | | | | | | | | | |
| ***1. General*** | 4 | 0.45 | [.35; 0.68] | 82% | .0905, p < .01 | ***1. General*** | 4 | 2.87 | [0.82; 9.99] | 95% | 1.5315, p < .01 |
| ***2. Youth/ college*** | 2 | 0.72 | [-.24; 1.68] | 79% | .3807, p = .03 | ***2. Youth/ college*** |  | – | – | – | – |
| ***3. Clinical*** | 1 | -0.26 | [-.56; .04] | – | – | ***3. Clinical*** | 2 | 1.65 | [0.49; 5.61] | 82% | 0.6440, p = .02 |
| ***4. IPV*** | 1 | 0.52 | [.35; .68] | – | – | ***4. IPV*** |  | – | – | – | – |
|  | ***Residual heterogeneity: 82%, p < .01*** | | | | |  | ***Residual heterogeneity: 94%, p < .01*** | | | | |
| **Study quality** | | | | | | | | | | | |
| ***1. Weak*** | 3 | 0.89 | [.35; 1.42] | 64% | 0.1393, p = .06 | ***1. Weak*** |  | – | – | – | – |
| ***2. Moderate*** | 3 | 0.18 | [-0.21; 0.58] | 90% | 0.1072, p < .01 | ***2.Moderate*** | 3 | 2.40 | [0.76; 7.58] | 87% | .8880,  p < .01 |
| ***3. Strong*** | 2 | 0.33 | [-0.12; 0.78] | 83% | 0.0875, p = .02 | ***3. Strong*** | 3 | 2.41 | [0.50; 11.63] | 96% | 0.8686, p < .01 |
|  | ***Residual heterogeneity: 84%, p < .01*** | | | | |  | ***Residual heterogeneity: 94%, p < .01*** | | | | |
