# Supplementary material for: The psychological subtype of intimate partner violence and its effect on mental health: a systematic review with meta-analyses
Source: Syst Rev. 2022 Aug 10;11:163. doi: 10.1186/s13643-022-02025-z (PMC9364557; doi:10.1186/s13643-022-02025-z)
Supplement: Supplementary file 5 — Additional file 5. Anxiety Subtype Analyses – Female Victimization [file 13643_2022_2025_MOESM5_ESM.docx]

| **Anxiety Subtype Analyses – Female Victimization** | | | | | | | | | | | |
| --- | --- | --- | --- | --- | --- | --- | --- | --- | --- | --- | --- |
| **Hedges g** | | | | | | **Odds Ratio** | | | | | |
|  | ***k*** | **Effect** | **95% CI** | **I^2^** | **τ^2^** |  | ***k*** | **OR** | **95% CI** | **I^2^** | **τ^2^** |
| **Scale measure** | | | | | | | | | | | |
| ***1. Valid IPV Scale*** | 17 | 0.55 | [0.33; 0.76] | 92% | 0.1773, p < .01 | ***1. Valid IPV Scale*** | 4 | 1.78 | [1.43; 2.22] | 23% | .0120, p = .27 |
| ***2. WHO etc.*** | 3 | 0.80 | [0.27; 1.33] | 86% | 0.1826, p < .01 | ***2. WHO etc.*** | 2 | 3.50 | [2.48; 4.94] | 0% | 0.0,  p = .70 |
|  |  |  |  |  |  | ***3. National item*** | 2 | 2.56 | [2.12; 3.09] | 0% | 0.0,  p = .40 |
|  | ***Residual heterogeneity: 92%, p < .01*** | | | | |  | ***Residual heterogeneity: %, p < .0*** | | | | |
| **Population** | | | | | | | | | | | |
| ***1. General*** | 8 | 0.53 | [0.23; 0.84] | 94% | 0.1811, p < .01 | ***1. General*** | 2 | 2.08 | [1.18; 3.67] | 29% | 0.6400, p = .23 |
| ***2. Youth/ college*** | 0 | – | – | – | – | ***2. Youth/ college*** | 0 | – | – | – | – |
| ***3. Clinical*** | 2 | 0.52 | [-0.20; 1.23] | 87% | 0.2308, p < .01 | ***3. Clinical*** | 10 | 2.22 | [1.24; 3.99] | 96% | 0.7064, p < .01 |
| ***4. IPV*** | 10 | 0.63 | [0.36; 0.90] | 90% | 0.1530, p < .01 | ***4. IPV*** | 0 | – | – | – | – |
|  | ***Residual heterogeneity: 92%, p < .01*** | | | | |  | ***Residual heterogeneity: 95%, p < .01*** | | | | |
| **Culture** | | | | | | | | | | | |
| ***1. EU*** | 3 | 0.81 | [0.27; 1.36] | 93% | 0.2807, p < .01 | ***1. EU*** | 2 | 1.68 | [0.68; 4.13] | 6% | 0.0567, p = .30 |
| ***2. US/AU*** | 10 | 0.49 | [0.25; 0.73] | 86% | 0.1276, p < .01 | ***2. US/AU*** | 6 | 2.26 | [1.05; 4.88] | 97% | 0.7838, p < .01 |
| ***3. South America*** |  | 0.58 | [-0.25; 1.41] | 98% | 0.3498, p < .01 | ***3. South America*** | 0 | – | – | – | – |
| ***4. Africa*** | 2 | 0.62 | [0.10; 1.13] | 94% | 0.2361, p < .01 | ***4. Africa*** | 4 | 2.09 | [0.97; 4.49] | 84% | 0.5113, p < .01 |
| ***5. Asia/ Middle East*** | 4 | 0.74 | [0.06; 1.42] | 91% | 0.2361, p < .01 | ***5. Asia/ Middle East*** | 0 | – | – | – | – |
|  | ***Residual heterogeneity: 93%, p < .01*** | | | | |  | ***Residual heterogeneity: 95%, p < .0*** | | | | |
| **Study quality** | | | | | | | | | | | |
| ***1. Weak*** | 9 | 0.70 | [0.38; 1.02] | 93% | 0.2363, p < .01 | ***1. Weak*** | 2 | 2.60 | [1.24; 5.45] | 0% | 0.0,  p = .30 |
| ***2. Moderate*** | 2 | 0.43 | [0.22; 0.65] | 89% | 0.0740, p < .01 | ***2.Moderate*** | 10 | 2.08 | [1.19; 3.64] | 96% | 0.6661, p < .01 |
| ***3. Strong*** | 8 | 0.51 | [-0.18; 1.20] | 87% | 0.2169, p < .01 | ***3. Strong*** | 0 | – | – | – | – |
|  | ***Residual heterogeneity: 91%, p < .01*** | | | | |  | ***Residual heterogeneity: 95%, p < .01*** | | | | |
