# Supplementary material for: The psychological subtype of intimate partner violence and its effect on mental health: a systematic review with meta-analyses
Source: Syst Rev. 2022 Aug 10;11:163. doi: 10.1186/s13643-022-02025-z (PMC9364557; doi:10.1186/s13643-022-02025-z)
Supplement: Supplementary file 6 — Additional file 6. References Used in Meta-Analyses [file 13643_2022_2025_MOESM6_ESM.docx]

**References Used in Meta-Analyses**

Ahmadabadi, Z., Najman, J. M., Williams, G. M., Clavarino, A. M., d'Abbs, P., & Tran, N. (2020). Intimate partner violence and subsequent depression and anxiety disorders. *Social psychiatry and psychiatric epidemiology., 07*.

Al-Modallal, H. (2012a). Psychological partner violence and women's vulnerability to depression, stress, and anxiety. *International Journal of Mental Health Nursing, 21*(6), 560-566. doi:10.1111/j.1447-0349.2012.00826.x

Al-Modallal, H., Sowan, A. K., Hamaideh, S., Peden, A. R., Al-Omari, H., & Al-Rawashdeh, A. B. (2012b). Psychological Outcomes of Intimate Partner Violence Experienced by Jordanian Working Women. *Health Care for Women International, 33*(3), 217-227. doi:10.1080/07399332.2011.610532

Albright, D. L., Fletcher, K. L., McDaniel, J., Thomas, K. H., Godfrey, K., Grohowski, M., & Dane, J. (2019). Intimate partner violence among postsecondary students with military experience. *Traumatology, 25*(1), 58-65. doi:http://dx.doi.org/10.1037/trm0000172

Alhalal, E., Ford-Gilboe, M., Wong, C., & Albuhairan, F. (2019). The Reliability and Validity of the Arabic Version of the Composite Abuse Scale. *Violence and Victims, 34*(1), 3-27. doi:10.1891/0886-6708.Vv-d-17-00111

Arias, I., Lyons, C. M., & Street, A. E. (1997). Individual and marital consequences of victimization: Moderating effects of relationship efficacy and spouse support. *Journal of Family Violence, 12*(2), 193-210. doi:10.1023/a:1022888728475

Babcock, J. C., Roseman, A., Green, C. E., & Ross, J. M. (2008). Intimate partner abuse and PTSD symptomatology: examining mediators and moderators of the abuse-trauma link. *Journal of Family Psychology, 22*(6), 809-818. doi:10.1037/a0013808

Baldry, A. C. (2003). "Stick and stones hurt my bones but his glance and words hurt more": The impact of physiological abuse and physical violence by current and former partners on battered women in Italy. *The International Journal of Forensic Mental Health, 2*(1), 47-57.

Barchi, F., Winter, S. C., Dougherty, D., Ramaphane, P., & Solomon, P. L. (2018). The Association of Depressive Symptoms and Intimate Partner Violence Against Women in Northwestern Botswana. *Journal of Interpersonal Violence*, 886260518792986. doi:10.1177/0886260518792986

Basile, K. C., Arias, I., Desai, S., & Thompson, M. P. (2004). The Differential Association of Intimate Partner Physical, Sexual, Psychological, and Stalking Violence and Posttraumatic Stress Symptoms in a Nationally Representative Sample of Women. *Journal of Traumatic Stress, 17*(5), 413-421.

Bebanic, V., Clench-Aas, J., Raanaas, R. K., & Nes, R. B. (2017). The Relationship Between Violence and Psychological Distress Among Men and Women: Do Sense of Mastery and Social Support Matter? *Journal of Interpersonal Violence, 32*(16), 2371-2395. doi:10.1177/0886260515591978

Beck, J., McNiff, J., Clapp, J. D., Olsen, S. A., Avery, M. L., & Hagewood, J. (2011). Exploring negative emotion in women experiencing intimate partner violence: Shame, Guilt, and PTSD. *Behavior Therapy, 42*(4), 740-750.

Becker, K. D., Stuewig, J., & McCloskey, L. A. (2010). Traumatic stress symptoms of women exposed to different forms of childhood victimization and intimate partner violence. *Journal of Interpersonal Violence, 25*(9), 1699-1715. doi:10.1177/0886260509354578

Blasco-Ros, C., Sanchez-Lorente, S., & Martinez, M. (2010). Recovery from depressive symptoms, state anxiety and post-traumatic stress disorder in women exposed to physical and psychological, but not to psychological intimate partner violence alone: A longitudinal study. *Bmc Psychiatry, 10*.

Calvete, E., Corral, S., & Estevez, A. (2007b). Cognitive and coping mechanisms in the interplay between intimate partner violence and depression. *Anxiety Stress Coping, 20*(4), 369-382. doi:10.1080/10615800701628850

Calvete, E., Corral, S., & Estvez, A. (2008). Coping as a mediator and moderator between intimate partner violence and symptoms of anxiety and depression. *Violence Against Women, 14*(8), 886-903.

Chandra, P. S., Satyanarayana, V. A., & Carey, M. P. (2009). Women reporting intimate partner violence in India: associations with PTSD and depressive symptoms. *Archives of Womens Mental Health, 12*(4), 203-209. doi:10.1007/s00737-009-0065-6

Chen, P.-H., Rovi, S., Vega, M., Jacobs, A., & Johnson, M. S. (2009). Relation of domestic violence to health status among Hispanic women. *Journal of Health Care for the Poor and Underserved, 20*(2), 569-582.

Cheung, D. S. T., Tiwari, A., Chan, K. L., Fong, D. Y. T., Chau, P. H., Yuen, F. K. H., & Tolman, R. M. (2017). Validation of the Psychological Maltreatment of Women Inventory for Chinese Women. *Journal of Interpersonal Violence*, 886260517715602. doi:10.1177/0886260517715602

Choi, D. W., Han, K. T., Jeon, J., Ju, Y. J., & Park, E. C. (2020). Association between family conflict resolution methods and depressive symptoms in South Korea: a longitudinal study. *Archives of Womens Mental Health, 23*(1), 123-129. doi:10.1007/s00737-019-00957-5

Chowdhary, N., & Patel, V. (2008). The effect of spousal violence on women's health: findings from the Stree Arogya Shodh in Goa, India. *Journal of Postgraduate Medicine, 54*(4), 306-312.

Coker, A. L., Weston, R., Creson, D. L., Justice, B., & Blakeney, P. (2005). PTSD Symptoms Among Men and Women Survivors of Intimate Partner Violence: The Role of Risk and Protective Factors. *Violence and Victims, 20*(6), 625-643. doi:10.1891/0886-6708.20.6.625

Comecanha, R., Basto-Pereira, M., & Maia, A. (2017). Clinically speaking, psychological abuse matters. *Comprehensive Psychiatry, 73*, 120-126. doi:10.1016/j.comppsych.2016.11.015

Cook, S. L., & Goodman, L. A. (2006). Beyond frequency and severity - Development and validation of the Brief Coercion and Conflict Scales. *Violence Against Women, 12*(11), 1050-1072. doi:10.1177/1077801206293333

Cowden, R. G., Worthington, E. L., Griffin, B. J., & Garthe, R. C. (2019). Forgiveness moderates relations between psychological abuse and indicators of psychological distress among women in romantic relationships. *South African Journal of Science, 115*(11-12). doi:10.17159/sajs.2019/6353

Dardis, C. M., Strauss, C. V., & Gidycz, C. A. (2019). The Psychological Toll of Unwanted Pursuit Behaviors and Intimate Partner Violence on Undergraduate Women: A Dominance Analysis. *Psychology of Violence, 9*(2), 209-220. doi:10.1037/vio0000189

de Mendoza. V. B., Harville, E. W., Savage, J., & Giarratano, G. (2018). Experiences of Intimate Partner and Neighborhood Violence and Their Association With Mental Health in Pregnant Women. *Journal of Interpersonal Violence, 33*(6), 938-959. doi:10.1177/0886260515613346

del Rio, I. D., & Valle, E. S. G. (2017). The Consequences of Intimate Partner Violence on Health: A Further Disaggregation of Psychological ViolenceEvidence From Spain. *Violence Against Women, 23*(14), 1771-1789. doi:10.1177/1077801216671220

DePrince, A. P., Buckingham, S. E., & Belknap, J. (2014). The geography of intimate partner abuse experiences and clinical responses. *Clinical Psychological Science, 2*(3), 258-271.

Desmarais, S. L., Pritchard, A., Lowder, E. M., & Janssen, P. A. (2014). Intimate partner abuse before and during pregnancy as risk factors for postpartum mental health problems. *BMC Pregnancy and Childbirth, 14 (1) (no pagination)*(132).

Dichter, M. E., Marcus, S. C., Wagner, C., & Bonomi, A. E. (2014). Associations between psychological, physical, and sexual intimate partner violence and health outcomes among women veteran VA patients. *Social Work in Mental Health, 12*(5-6), 411-428.

dos Santos, A. G., & Monteiro, C. F. D. (2018). Domains of common mental disorders in women reporting intimate partner violence. *Revista Latino-Americana De Enfermagem, 26*. doi:10.1590/1518-8345.2740.3099

Duerksen, K. N., & Woodin, E. M. (2019). Cyber Dating Abuse Victimization: Links With Psychosocial Functioning. *Journal of Interpersonal Violence*. doi:10.1177/0886260519872982

Ely, G. E., & Otis, M. D. (2011). An Examination of Intimate Partner Violence and Psychological Stressors in Adult Abortion Patients. *Journal of Interpersonal Violence, 26*(16), 3248-3266. doi:10.1177/0886260510393004

Esie, P., Osypuk, T. L., Schuler, S. R., & Bates, L. M. (2019). Intimate partner violence and depression in rural Bangladesh: Accounting for violence severity in a high prevalence setting. *SSM - Population Health, 7 (no pagination)*(100368).

Estefan, L. F., Coulter, M. L., & VandeWeerd, C. (2016). Depression in Women Who Have Left Violent Relationships: The Unique Impact of Frequent Emotional Abuse. *Violence Against Women, 22*(11), 1397-1413. doi:10.1177/1077801215624792

Fahmy, H. H., & Abd El-Rahman, S. I. (2008). Determinants and health consequences of domestic violence among women in reproductive age at zagazig district, egypt. *Journal of Egyptian Public Health Association, 83*(1-2), 87-106.

Follingstad, D. R., & Rogers, M. J. (2012). Women experiencing psychological abuse: Are they a homogenous group? *Journal of Aggression, Maltreatment and Trauma, 21*(8), 891-916.

Garcia-Linares, M., Sanchez-Lorente, S., Coe, C. L., & Martinez, M. (2004). Intimate Male Partner Violence Impairs Immune Control Over Herpes Simplex Virus Type 1 in Physically and Psychologically Abused Women. *Psychosomatic Medicine, 66*(6), 965-972.

Gobin, R. L., Iverson, K. M., Mitchell, K., Vaughn, R., & Resick, P. A. (2013). The impact of childhood maltreatment on PTSD symptoms among female survivors of intimate partner violence. *Violence and Victims, 28*(6), 984-999.

Grandin, E., Lupri, E., & Brinkerhoff, M. B. (1998). Couple violence and psychological distress. *Canadian Journal of Public Health, 89*(1), 43-47.

Greene, C. A., Chan, G., McCarthy, K. J., Wakschlag, L. S., & Briggs-Gowan, M. J. (2018). Psychological and physical intimate partner violence and young children's mental health: The role of maternal posttraumatic stress symptoms and parenting behaviors. *Child Abuse & Neglect, 77*, 168-179. doi:10.1016/j.chiabu.2018.01.012

Gucek, N. K., & Selic, P. (2018). Depression in Intimate Partner Violence Victims in Slovenia: A Crippling Pattern of Factors Identified in Family Practice Attendees. *International Journal of Environmental Research and Public Health, 15*(2). doi:10.3390/ijerph15020210

Haj-Yahia, M. M. (2000a). Patterns of violence against engaged Arab women from Israel and some psychological implications. *Psychology of Women Quarterly, 24*(3), 209-219.

Haj-Yahia, M. M. (2000b). Implications of wife abuse and battering for self-esteem, depression, and anxiety as revealed by the Second Palestinian National Survey on Violence Against Women. *Journal of Family Issues, 21*(4), 435-463.

Han, K. M., Jee, H. J., An, H., Shin, C., Yoon, H. K., Ko, Y. H., . . . Han, C. (2019). Intimate partner violence and incidence of depression in married women: A longitudinal study of a nationally representative sample. *Journal of Affective Disorders, 245*, 305-311. doi:10.1016/j.jad.2018.11.041

Hansrod, F., Spies, G., & Seedat, S. (2015). Type and severity of intimate partner violence and its relationship with PTSD in HIV-infected women. *Psychology Health & Medicine, 20*(6), 697-709. doi:10.1080/13548506.2014.967702

Hassan, S., & Malik, A. A. (2012). Psycho-social correlates of intimate partner violence. *Pakistan Journal of Psychological Research, 27*(2), 279-295.

Hedin, L. W., & Janson, P. O. (1999). The invisible wounds: the occurrence of psychological abuse and anxiety compared with previous experience of physical abuse during the childbearing year. *Journal of Psychosomatic Obstetrics & Gyneocology, 20*(3), 136-144.

Hegarty, K., Gunn, J., Chondros, P., & Small, R. (2004). Association between depression and abuse by partners of women attending general practice: descriptive, cross sectional survey. *British Medical Journal, 328*(7440), 621-624. doi:10.1136/bmj.328.7440.621

Hegarty, K. L., O'Doherty, L. J., Chondros, P., Valpied, J., Taft, A. J., Astbury, J., . . . Gunn, J. M. (2013). Effect of type and severity of intimate partner violence on women's health and service use: findings from a primary care trial of women afraid of their partners. *Journal of Interpersonal Violence, 28*(2), 273-294. doi:10.1177/0886260512454722

Hicks, M. H., & Li, Z. (2003). Partner violence and major depression in women: a community study of Chinese Americans. *Journal of Nervous Mental Disease, 191*(11), 722-729. doi:10.1097/01.nmd.0000095124.05023.e1

Hines, D. A., & Douglas, E. M. (2011). Symptoms of posttraumatic stress disorder in men who sustain intimate partner violence: A study of helpseeking and community samples. *Psychology of Men & Masculinity, 12*(2), 112-127.

Hines, D. A., & Douglas, E. M. (2016). Relative Influence of Various Forms of Partner Violence on the Health of Male Victims: Study of a Helpseeking Sample. *Psychology of Men & Masculinity, 17*(1), 3-16. doi:10.1037/a0038999

Holmes, S. C., Johnson, N. L., Zlotnick, C., Sullivan, T. P., & Johnson, D. M. (2020). The Association Between Demographic, Mental Health, and Intimate Partner Violence Victimization Variables and Undergraduate Women's Intimate Partner Violence Perpetration. *Journal of Interpersonal Violence*. doi:10.1177/0886260520907354

Houry, D., Kemball, R., Rhodes, K. V., & Kaslow, N. J. (2006). Intimate partner violence and mental health symptoms in African American female ED patients. *American Journal Emergency Medicine, 24*(4), 444-450. doi:10.1016/j.ajem.2005.12.026

Huang, C. J., & Gunn, T. (2001). An examination of domestic violence in an African American community in North Carolina: Causes and consequences. *Journal of Black Studies, 31*(6), 790-811.

Huth-Bocks, A. C., Krause, K., Ahlfs-Dunn, S., Gallagher, E., & Scott, S. (2013). Relational trauma and posttraumatic stress symptoms among pregnant women. *Psychodynamic Psychiatry, 41*(2), 277-301. doi:10.1521/pdps.2013.41.2.277

Jaquier, V., Flanagan, J. C., & Sullivan, T. P. (2015). Anxiety and posttraumatic stress symptom pathways to substance use problems among community women experiencing intimate partner violence. *Anxiety, Stress & Coping: An International Journal, 28*(4), 445-455.

Jeter, W. K., & Brannon, L. A. (2014). Moving Beyond "Sticks and Stones": Chronic Psychological Trauma Predicts Posttraumatic Stress Symptoms. *Journal of Trauma & Dissociation, 15*(5), 548-556. doi:10.1080/15299732.2014.907596

Jina, R., Jewkes, R., Hoffman, S., Dunkle, K. L., Nduna, M., & Shai, N. J. (2012). Adverse mental health outcomes associated with emotional abuse in young rural South African women: A cross-sectional study. *Journal of Interpersonal Violence, 27*(5), 862-880.

Jonas, S., Khalifeh, H., Bebbington, P. E., McManus, S., Brugha, T., Meltzer, H., & Howard, L. M. (2014). Gender differences in intimate partner violence and psychiatric disorders in England: results from the 2007 adult psychiatric morbidity survey. *Epidemiology and Psychiatric Sciences, 23*(2), 189-199. doi:10.1017/s2045796013000292

Jones, S., Davidson, W. S., Bogat, G. A., Levendosky, A., & von Eye, A. (2005). Validation of the Subtle and Overt Psychological Abuse Scale: An Examination of Construct Validity. *Violence and Victims*(4), 407-416. doi:10.1891/0886-6708.20.4.407

Katz, J., & Arias, I. (1999). Psychological abuse and depressive symptoms in dating women: Do different types of abuse have differential effects? *Journal of Family Violence, 14*(3), 281-295.

Kelly, U. A. (2010b). Symptoms of PTSD and major depression in Latinas who have experienced intimate partner violence. *Issues in Mental Health Nursing, 31*(2), 119-127. doi:10.3109/01612840903312020

Kiene, S. M., Lule, H., Sileo, K. M., Silmi, K. P., & Wanyenze, R. K. (2017). Depression, alcohol use, and intimate partner violence among outpatients in rural Uganda: vulnerabilities for HIV, STIs and high risk sexual behavior. *Bmc Infectious Diseases, 17*. doi:10.1186/s12879-016-2162-2

Kinyanda, E., Weiss, H. A., Mungherera, M., Onyango-Mangen, P., Ngabirano, E., Kajungu, R., . . . Patel, V. (2016). Intimate partner violence as seen in post-conflict eastern Uganda: prevalence, risk factors and mental health consequences. *Bmc International Health and Human Rights, 16*. doi:10.1186/s12914-016-0079-x

Koopman, C., Ismailji, T., Palesh, O., Gore-Felton, C., Narayanan, A., Saltzman, K. M., . . . McGarvey, E. L. (2007). Relationships of depression to child and adult abuse and bodily pain among women who have experienced intimate partner violence. *Journal of Interpersonal Violence, 22*(4), 438-455. doi:10.1177/0886260506297028

Lacey, K. K., McPherson, M. D., Samuel, P. S., Sears, K. P., & Head, D. (2013). The Impact of Different Types of Intimate Partner Violence on the Mental and Physical Health of Women in Different Ethnic Groups. *Journal of Interpersonal Violence, 28*(2), 359-385. doi:10.1177/0886260512454743

Levine, A. R., & Fritz, P. A. (2016). Coercive control, posttraumatic stress disorder, and depression among homeless women. *Partner Abuse, 7*(1), 26-43.

Lilly, M. M., & Graham-Bermann, S. A. (2010). Intimate Partner Violence and PTSD: The Moderating Role of Emotion-Focused Coping. *Violence and Victims*(5), 604-616. doi:10.1891/0886-6708.25.5.604

Longares, L., Escartin, J., & Rodriguez-Carballeira, A. (2016). Collective Self-Esteem and Depressive Symptomatology in Lesbians and Gay Men: A Moderated Mediation Model of Self-Stigma and Psychological Abuse. *Journal of Homosexuality*. doi:10.1080/00918369.2016.1223333

Longares, L., Saldana, O., Escartin, J., Barrientos, J., & Rodriguez-Carballeira, A. (2018). Measuring psychological abuse in same-sex couples: evidence of validity of the EAPA-P in a Spanish-speaking sample. *Anales De Psicologia, 34*(3), 555-561. doi:10.6018/analesps.34.3.306281

Lovestad, S., Love, J., Vaez, M., & Krantz, G. (2017). Prevalence of intimate partner violence and its association with symptoms of depression; a cross-sectional study based on a female population sample in Sweden. *BMC Public Health, 17*(1), 335. doi:10.1186/s12889-017-4222-y

Martin, S. L., Li, Y., Casanueva, C., Harris-Britt, A., Kupper, L. L., & Cloutier, S. (2006). Intimate partner violence and women's depression before and during pregnancy. *Violence Against Women, 12*(3), 221-239. doi:10.1177/1077801205285106

Matseke G, Rodriguez VJ, Peltzer K, Jones D. Intimate partner violence among HIV positive pregnant women in South Africa. Journal of Psychology in Africa. 2016;26(3):259-66.

Matud, M. (2005). The psychological impact of domestic violence on Spanish women. *Journal of Applied Social Psychology, 35*(11), 2310-2322.

McMahon, S., Huang, C. C., Boxer, P., & Postmus, J. L. (2011). The impact of emotional and physical violence during pregnancy on maternal and child health at one year post-partum. *Children and Youth Services Review, 33*(11), 2103-2111. doi:10.1016/j.childyouth.2011.06.001

Mechanic, M. B., Uhlmansiek, M. H., Weaver, T. L., & Resick, P. A. (2000). The Impact of Severe Stalking Experienced by Acutely Battered Women: An Examination of Violence, Psychological Symptoms and Strategic Responding. *Violence and Victims*(4), 443-458. doi:10.1891/0886-6708.15.4.443

Mechanic, M. B., Weaver, T. L., & Resick, P. A. (2008). Mental health consequences of intimate partner abuse: a multidimensional assessment of four different forms of abuse. *Violence Against Women, 14*(6), 634-654. doi:10.1177/1077801208319283

Melander, L. A., & Marganski, A. J. (2020). Cyber and In-Person Intimate Partner Violence Victimization: Examining Maladaptive Psychosocial and Behavioral Correlates. *Cyberpsychology-Journal of Psychosocial Research on Cyberspace, 14*(1). doi:10.5817/cp2020-1-1

Miller, B., & Irvin, J. (2017). Invisible Scars: Comparing the Mental Health of LGB and Heterosexual Intimate Partner Violence Survivors. *Journal of Homosexuality, 64*(9), 1180-1195. doi:10.1080/00918369.2016.1242334

Miller-Graff, L. E., & Cheng, P. (2017). Consequences of Violence Across the Lifespan: Mental Health and Sleep Quality in Pregnant Women. *Psychological Trauma-Theory Research Practice and Policy, 9*(5), 587-595. doi:10.1037/tra0000252

Mills, C. P., Hill, H. M., & Johnson, J. A. (2018). Mediated effects of coping on mental health outcomes of African American women exposed to physical and psychological abuse. *Violence Against Women, 24*(2), 186-206.

Mittal, M., Resch, K., Nichols-Hadeed, C., Stone, J. T., Thevenet-Morrison, K., Faurot, C., & Cerulli, C. (2018). Examining Associations Between Strangulation and Depressive Symptoms in Women With Intimate Partner Violence Histories. *Violence and Victims, 33*(6), 1072-1087. doi:10.1891/0886-6708.Vv-d-16-00223

Montgomery, B. E., Rompalo, A., Hughes, J., Wang, J., Haley, D., Soto-Torres, L., . . . Hodder, S. (2015). Violence Against Women in Selected Areas of the United States. *American Journal of Public Health, 105*(10), 2156-2166. doi:10.2105/ajph.2014.302430

Mouton, C. P., Rodabough, R. J., Rovi, S. L. D., Brzyski, R. G., & Katerndahl, D. A. (2010). Psychosocial Effects of Physical and Verbal Abuse in Postmenopausal Women. *Annals of Family Medicine, 8*(3), 206-213. doi:10.1370/afm.1095

Mugoya, G. C. T., Mumba, M. N., Hooper, L. M., Witte, T., & Youngblood, M. (2020). Depression and intimate partner violence among urban Kenyan caregivers of children with disabilities. *Journal of Psychiatric and Mental Health Nursing, 27*(1), 41-53. doi:10.1111/jpm.12550

Mugoya, G. C. T., Witte, T., Bolland, A., Tomek, S., Hooper, L. M., Bolland, J., & Dalmida, S. G. (2020). Depression and Intimate Partner Violence Among African American Women Living in Impoverished Inner-City Neighborhoods. *Journal of Interpersonal Violence, 35*(3-4), 899-923. doi:10.1177/0886260517691519

Nathanson, A. M., Shorey, R. C., Tirone, V., & Rhatigan, D. L. (2012). The Prevalence of Mental Health Disorders in a Community Sample of Female Victims of Intimate Partner Violence. *Partner Abuse, 3*(1), 59-75. doi:10.1891/1946-6560.3.1.59

Nedd, D. M. (2001). Self-reported health status and depression of battered black women. *The ABNF journal : official journal of the Association of Black Nursing Faculty in Higher Education, Inc, 12*(2), 32-35.

Nnawulezi, N., & Murphy, C. (2019). Understanding Formal Help-Seeking Among Women Whose Partners Are in Abuser Intervention Programs. *Psychology of Violence, 9*(4), 383-391. doi:10.1037/vio0000126

Nurius, P. S., Macy, R. J., Bhuyan, R., Holt, V. L., Kernic, M. A., & Rivara, F. P. (2003). Contextualizing Depression and Physical Functioning in Battered Women: Adding Vulnerability and Resources to the Analysis. *Journal of Interpersonal Violence, 18*(12), 1411-1431.

Overstreet, N. M., Willie, T. C., Hellmuth, J. C., & Sullivan, T. P. (2015). Psychological intimate partner violence and sexual risk behavior: examining the role of distinct posttraumatic stress disorder symptoms in the partner violence-sexual risk link. *Womens Health Issues, 25*(1), 73-78. doi:10.1016/j.whi.2014.10.005

Pantalone, D. W., Schneider, K. L., Valentine, S. E., & Simoni, J. M. (2012). Investigating Partner Abuse Among HIV-Positive Men Who have Sex with Men. *AIDS and Behavior, 16*(4), 1031-1043. doi:10.1007/s10461-011-0011-2

Panuzio, J., Taft, C. T., Black, D. A., Koenen, K. C., & Murphy, C. M. (2007). Relationship abuse and victims' posttraumatic stress disorder symptoms: Associations with child behavior problems. *Journal of Family Violence, 22*(4), 177-185.

Peltzer, K., & Pengpid, S. (2017). Associations between intimate partner violence, depression, and suicidal behavior among women attending antenatal and general outpatients hospital services in Thailand. *Nigerian Journal of Clinical Practice, 20*(7), 892-899. doi:10.4103/njcp.njcp_453_15

Pickover, A. M., Lipinski, A. J., Dodson, T. S., Tran, H. N., Woodward, M. J., & Beck, J. (2017). Demand/withdraw communication in the context of intimate partner violence: Implications for psychological outcomes. *Journal of Anxiety Disorders, 52*, 95-102.

Pico-Alfonso, M. A., Garcia-Linares, M., Celda-Navarro, N., Blasco-Ros, C., Echeburua, E., & Martinez, M. (2006). The Impact of Physical, Psychological, and Sexual Intimate Male Partner Violence on Women's Mental Health: Depressive Symptoms, Posttraumatic Stress Disorder, State Anxiety, and Suicide. *Journal of Women's Health, 15*(5), 599-611.

Postmus, J. L., Huang, C. C., & Mathisen-Stylianou, A. (2012). The impact of physical and economic abuse on maternal mental health and parenting. *Children and Youth Services Review, 34*(9), 1922-1928. doi:10.1016/j.childyouth.2012.06.005

Ratner, P. A. (1993). The incidence of wife abuse and mental health status in abused wives in Edmonton, Alberta. *Canadian Journal of Public Health, 84*(4), 246-249.

Rauer, A. J., & El-Sheikh, M. (2012). Reciprocal pathways between intimate partner violence and sleep in men and women. *Journal of Family Psychology, 26*(3), 470-477.

Rauer, A. J., Kelly, R. J., Buckhalt, J. A., & El-Sheikh, M. (2010). Sleeping with one eye open: marital abuse as an antecedent of poor sleep. *Journal of Family Psychology, 24*(6), 667-677. doi:10.1037/a0021354

Rees, S. J., Tol, W., Mohsin, M., Tay, A. K., Tam, N., dos Reis, N., . . . Silove, D. M. (2016). A high-risk group of pregnant women with elevated levels of conflict-related trauma, intimate partner violence, symptoms of depression and other forms of mental distress in post-conflict Timor-Leste. *Translational Psychiatry, 6*, e725. doi:10.1038/tp.2015.212

Reich, C. M., Jones, J. M., Woodward, M. J., Blackwell, N., Lindsey, L. D., & Beck, J. G. (2015). Does Self-Blame Moderate Psychological Adjustment Following Intimate Partner Violence? *Journal of Interpersonal Violence, 30*(9), 1493-1510. doi:10.1177/0886260514540800

Renner, L. M. (2009). Intimate Partner Violence Victimization and Parenting Stress: Assessing the Mediating Role of Depressive Symptoms. *Violence Against Women, 15*(11), 1380-1401. doi:10.1177/1077801209346712

Roh, S., Burnette, C. E., Lee, K. H., Lee, Y.-S., & Easton, S. D. (2016). Risk and protective factors for depressive symptoms among indigenous older adults: Intimate partner violence (IPV) and social support. *Journal of Gerontological Social Work, 59*(4), 316-331.

Rurangirwa, A. A., Mogren, I., Ntaganira, J., Govender, K., & Krantz, G. (2018). Intimate partner violence during pregnancy in relation to non-psychotic mental health disorders in Rwanda: A cross-sectional population-based study. *BMJ Open, 8 (7) (no pagination)*(e021807).

Sabri, B., Bolyard, R., McFadgion, A. L., Stockman, J. K., Lucea, M. B., Callwood, G. B., . . . Campbell, J. C. (2013b). Intimate partner violence, depression, PTSD, and use of mental health resources among ethnically diverse Black women. *Social Work in Health Care, 52*(4), 351-369.

Sackett, L. A., & Saunders, D. G. (1999). The impact of different forms of psychological abuse on battered women. *Violence and Victims, 14*(1), 105-117.

Sargent, K. S., Krauss, A., Jouriles, E. N., & McDonald, R. (2016). Cyber Victimization, Psychological Intimate Partner Violence, and Problematic Mental Health Outcomes Among First-Year College Students. *Cyberpsychology Behavior and Social Networking, 19*(9), 545-550. doi:10.1089/cyber.2016.0115

Sauber, E. W., & O'Brien, K. M. (2017). Multiple Losses: The Psychological and Economic Well-Being of Survivors of Intimate Partner Violence. *Journal of Interpersonal Violence*, 886260517706760. doi:10.1177/0886260517706760

Shamu, S., Zarowsky, C., Roelens, K., Temmerman, M., & Abrahams, N. (2016). High-frequency intimate partner violence during pregnancy, postnatal depression and suicidal tendencies in Harare, Zimbabwe. *General Hospital Psychiatry, 38*, 109-114.

Shen, A. C.-T. (2014). Dating violence and posttraumatic stress disorder symptoms in Taiwanese college students: The roles of cultural beliefs. *Journal of Interpersonal Violence, 29*(4), 635-658.

Shen, S., & Kusunoki, Y. (2019). Intimate Partner Violence and Psychological Distress Among Emerging Adult Women: A Bidirectional Relationship. *Journal of Womens Health, 28*(8), 1060-1067. doi:10.1089/jwh.2018.7405

Shorey, R. C., Febres, J., Brasfield, H., & Stuart, G. L. (2012). Male dating violence victimization and adjustment: the moderating role of coping. *American Journal of Mens Health, 6*(3), 218-228. doi:10.1177/1557988311429194

Signorelli, M. S., Arcidiacono, E., & Aguglia, E. (2012). What factors are associated with intimate partner violence? Evaluation of the impact of risk factors and psychopathological consequences. *European Psychiatry. Conference: 20th European Congress of Psychiatry, EPA, 27*(SUPPL. 1).

Simonelli, C. J., & Ingram, K. M. (1998). Psychological distress among men experiencing physical and emotional abuse in heterosexual dating relationships. *Journal of Interpersonal Violence, 13*(6), 667-681.

Smagur, K. E., Bogat, G. A., & Levendosky, A. A. (2018). Attachment Insecurity Mediates the Effects of Intimate Partner Violence and Childhood Maltreatment on Depressive Symptoms in Adult Women. *Psychology of Violence, 8*(4), 460-469. doi:10.1037/vio0000142

Sotskova, A., & Woodin, E. M. (2013). Posttraumatic Stress, Partner Violence Victimization, and Harmful Drinking: Risk Factors for Relationship Discord in New Parents. *Journal of Interpersonal Violence, 28*(17), 3319-3341. doi:10.1177/0886260513496896

Street, A. E., & Arias, I. (2001). Psychological abuse and posttraumatic stress disorder in battered women: Examining the roles of shame and guilt. *Violence and Victims, 16*(1), 65-78.

Stylianou, A. M. (2018). Economic abuse experiences and depressive symptoms among victims of intimate partner violence. *Journal of Family Violence*, No Pagination Specified.

Sullivan, T. P., Cavanaugh, C. E., Buckner, J. D., & Edmondson, D. (2009). Testing posttraumatic stress as a mediator of physical, sexual, and psychological intimate partner violence and substance problems among women. *Journal of Traumatic Stress, 22*(6), 575-584.

Sullivan, T. P., McPartland, T., Price, P., Cruza-Guet, M. C., & Swan, S. C. (2013). Relationship self-Efficacy protects against mental health problems among women in bidirectionally aggressive intimate relationships with men. *Journal of Counseling Psychology, 60*(4), 641-647.

Tadegge, A. D. (2008). The mental health consequences of intimate partner violence against women in AgaroTown, southwest Ethiopia. *Tropical Doctor, 38*(4), 228-229. doi:10.1258/td.2008.070353

Tang, C. S.-K. (1998). Psychological abuse of Chinese wives. *Journal of Family Violence, 13*(3), 299-314.

Tang, C. S. K. (1997). Psychological impact of wife abuse - Experiences of Chinese women and their children. *Journal of Interpersonal Violence, 12*(3), 466-478. doi:10.1177/088626097012003010

Theran, S. A., Sullivan, C. M., Bogat, G., & Stewart, C. S. (2006). Abusive Partners and Ex-Partners: Understanding the Effects of Relationship to the Abuser on Women's Well-Being. *Violence Against Women, 12*(10), 950-969.

Thompson, M. P., Kaslow, N. J., Kingree, J. B., Puett, R., Thompson, N. J., & Meadows, L. (1999). Partner abuse and posttraumatic stress disorder as risk factors for suicide attempts in a sample of low-income, inner-city women. *Journal of Traumatic Stress, 12*(1), 59-72. doi:10.1023/a:1024742215337

Thompson, M. P., Kaslow, N. J., Kingree, J. B., Rashid, A., Puett, R., Jacobs, D., & Matthews, A. (2000). Partner violence, social support, and distress among inner-city African American women. *American Journal of Community Psychology, 28*(1), 127-143. doi:10.1023/a:1005198514704

Tiwari, A., Fong, D. Y., Chan, C.-H., & Ho, P.-C. (2013). Factors mediating the relationship between intimate partner violence and chronic pain in Chinese women. *Journal of Interpersonal Violence, 28*(5), 1067-1087.

Torres, S., & Han, H. R. (2000). Psychological distress in non-Hispanic White and Hispanic abused women. *Archives of Psychiatric Nursing, 14*(1), 19-29. doi:10.1016/s0883-9417(00)80005-9

Torres, S. N. (2019). The impact of coping self-efficacy and event centrality on PTSD symptom severity and posttraumatic growth among survivors of IPV. *Dissertation Abstracts International: Section B: The Sciences and Engineering, 80*(1-B(E)), No Pagination Specified.

Tung, I., Keenan, K., Stepp, S. D., & Hipwell, A. E. (2019). The moderating effects of traumatic stress on vulnerability to emotional distress during pregnancy. *Development and Psychopathology*, No Pagination Specified. doi:http://dx.doi.org/10.1017/S0954579419000531

Tyson, S. Y., Herting, J. R., & Randell, B. P. (2007). Beyond violence: Threat reappraisal in women recently separated from intimate-partner violent relationships. *Journal of Social and Personal Relationships, 24*(5), 693-706. doi:10.1177/0265407507081455

Umubyeyi, A., Mogren, I., Ntaganira, J., & Krantz, G. (2014). Intimate partner violence and its contribution to mental disorders in men and women in the post genocide Rwanda: findings from a population based study. *Bmc Psychiatry, 14*. doi:10.1186/s12888-014-0315-7

Vargas, A. E. O., & Tucker, M. S. (2015). Risk factors related to maternal self-efficacy among Mexican women: A comparison between Mexico and the United States of America. *Anuario de Psicologia, 45*(1), 25-38.

Varma, D., Chandra, P. S., Thomas, T., & Carey, M. P. (2007). Intimate partner violence and sexual coercion among pregnant women in India: relationship with depression and post-traumatic stress disorder. *Journal of Affective Disorders, 102*(1-3), 227-235. doi:10.1016/j.jad.2006.09.026

Wangel, A. M., Ryding, E. L., Schei, B., Ostman, M., Lukasse, M., & Bidens Study, G. (2016). Emotional, physical, and sexual abuse and the association with symptoms of depression and posttraumatic stress in a multi-ethnic pregnant population in southern Sweden. *Sexual & Reproductive Healthcare, 9*, 7-13. doi:10.1016/j.srhc.2016,04.003

Weiss, N. H., Dixon-Gordon, K. L., Duke, A. A., & Sullivan, T. P. (2015). The underlying role of posttraumatic stress disorder symptoms in the association between intimate partner violence and deliberate self-harm among African American women. *Comprehensive Psychiatry, 59*, 8-16.

Wijma, K., Samelius, L., Wingren, G., & Wijma, B. (2007). The association between ill-health and abuse: A cross-sectional population based study. *Scandinavian Journal of Psychology, 48*(6), 567-575.

Wolford-Clevenger C, Vann NC, Smith PN. The Association of Partner Abuse Types and Suicidal Ideation Among Men and Women College Students. Violence and Victims. 2016;31(3):471-85.

Wolford-Clevenger, C., & Smith, P. N. (2017a). The Conditional Indirect Effects of Suicide Attempt History and Psychiatric Symptoms on the Association Between Intimate Partner Violence and Suicide Ideation. *Personality Individual Differences, 106*, 46-51. doi:10.1016/j.paid.2016.10.042

Woods, S. J. (2000). Prevalence and patterns of posttraumatic stress disorder in abused and postabused women. *Issues in Mental Health Nursing, 21*(3), 309-324.

Woods, S. J., Hall, R. J., Campbell, J. C., & Angott, D. M. (2008). Physical health and posttraumatic stress disorder symptoms in women experiencing intimate partner violence. *Journal of Midwifery & Women's Health, 53*(6), 538-546.

Woods, S. J., Kozachik, S. L., & Hall, R. J. (2010). Subjective Sleep Quality in Women Experiencing Intimate Partner Violence: Contributions of Situational, Psychological, and Physiological Factors. *Journal of Traumatic Stress, 23*(1), 141-150. doi:10.1002/jts.20495

Wuest, J., Ford-Gilboe, M., Merritt-Gray, Wilk, P., Campbell, J., C., Lent, B., . . . Smye, V. (2010). Pathways of chronic pain in survivors of intimate partner violence. *Journal of Women's Health, 19*(9), 1665-1674.

Yuan, W. M., & Hesketh, T. (2019). Intimate Partner Violence and Depression in Women in China. *Journal of Interpersonal Violence*. doi:10.1177/0886260519888538

Zacarias, A. E., Macassa, G., Soares, J. J. F., Svanstrom, L., & Antai, D. (2012). Symptoms of depression, anxiety, and somatization in female victims and perpetrators of intimate partner violence in Maputo City, Mozambique. *International Journal of Women's Health, 4*(1), 491-503.
